# Metabolic Pathway Assignment of Plant Genes based on Phylogenetic Profiling–A Feasibility Study

**DOI:** 10.3389/fpls.2017.01831

**Published:** 2017-10-27

**Authors:** Sandra Weißenborn, Dirk Walther

**Affiliations:** Max Planck Institute of Molecular Plant Physiology, Potsdam, Germany

**Keywords:** phylogenetic profiling, plants, metabolic pathways, secondary metabolism, gene families, gene function annotation, protein-protein interactions, gene co-expression

## Abstract

Despite many developed experimental and computational approaches, functional gene annotation remains challenging. With the rapidly growing number of sequenced genomes, the concept of phylogenetic profiling, which predicts functional links between genes that share a common co-occurrence pattern across different genomes, has gained renewed attention as it promises to annotate gene functions based on presence/absence calls alone. We applied phylogenetic profiling to the problem of metabolic pathway assignments of plant genes with a particular focus on secondary metabolism pathways. We determined phylogenetic profiles for 40,960 metabolic pathway enzyme genes with assigned EC numbers from 24 plant species based on sequence and pathway annotation data from KEGG and Ensembl Plants. For gene sequence family assignments, needed to determine the presence or absence of particular gene functions in the given plant species, we included data of all 39 species available at the Ensembl Plants database and established gene families based on pairwise sequence identities and annotation information. Aside from performing profiling comparisons, we used machine learning approaches to predict pathway associations from phylogenetic profiles alone. Selected metabolic pathways were indeed found to be composed of gene families of greater than expected phylogenetic profile similarity. This was particularly evident for primary metabolism pathways, whereas for secondary pathways, both the available annotation in different species as well as the abstraction of functional association via distinct pathways proved limiting. While phylogenetic profile similarity was generally not found to correlate with gene co-expression, direct physical interactions of proteins were reflected by a significantly increased profile similarity suggesting an application of phylogenetic profiling methods as a filtering step in the identification of protein-protein interactions. This feasibility study highlights the potential and challenges associated with phylogenetic profiling methods for the detection of functional relationships between genes as well as the need to enlarge the set of plant genes with proven secondary metabolism involvement as well as the limitations of distinct pathways as abstractions of relationships between genes.

## Introduction

Developing an understanding of plant metabolism is a central aim of plant research. The better we can assess the metabolic capacities of plants and how they regulate their metabolic activities, the better we can make use of the manifold of products and also protect their fragile ecosystems. In principle, it should be possible to estimate a plant's metabolic capacity based on the knowledge of all possible metabolic reactions that are in turn encoded by the repertoire of enzyme genes in the respective genome. Thus, complete and accurate genome annotation is paramount for a comprehensive understanding of plant metabolism. However, reliable functional gene annotation is neither trivial nor is our current knowledge of possible metabolic pathways complete. We are not yet able to simply check for the presence of “textbook pathways” by virtue of accurate gene annotation. Novel pathways, in particular in the context of secondary metabolite pathways, are still being discovered, requiring, however, substantial experimental effort as demonstrated in the discovery of a strigolactone pathway in plants (Alder et al., 2012).

Considering the high costs and immense efforts of experimental gene function annotation, computational comparative genomics remains the main strategy to assign functions to genes in plants. Established functional assignment methods aim to bioinformatically predict functions of proteins of not yet annotated species by searching for sequence-similar proteins carrying reliably annotated, ideally experimentally verified, functions. Assuming that high sequence similarity assures similarity of function, functional annotations are transferred from the characterized to the new and yet uncharacterized gene (Lohse et al., 2014).

However, genes active in the same biochemical pathway will typically perform distinct enzymatic functions and thus will generally not show any amino acid sequence similarity to each other, albeit a weak, but discernable correlation between metabolic pathway and enzyme protein sequence distances has been reported pointing to a gradual expansion of metabolism (Schutte et al., 2010). Notwithstanding this observation, sequence-homology-based methods are generally of limited use for generating connections between components that perform different functions as part of the same pathway. Homology-based functional prediction methods are also inherently limited by relying upon the set of known functional annotations.

Several strategies have been developed to establish functional links between genes performing different functions. They rely on the observed physical proximity of pathway-associated genes originating from bacterial operon genome structures (Osbourn, 2010; Chu et al., 2011) or the pronounced co-expression of genes (Gachon et al., 2005; Wisecaver et al., 2016). Genome-wide association studies allow identifying genes commonly participating in, or regulating, the biosynthesis of a particular metabolite (Yencho et al., 1998; Schauer et al., 2006; Kliebenstein, 2009).

Phylogenetic profiling offers yet another approach to detect functional gene associations. Phylogenetic profiling was developed based on the notion that genes involved in the same metabolic pathway, or generally are involved in the same functional process, are likely to evolve in a correlated fashion (Gaasterland and Ragan, 1998; Pellegrini et al., 1999). For a given process, all its essential elements (genes) are either present—as they are all needed to perform a particular function—, or are all absent, because if any component is absent, all other components can no longer function lifting the evolutionary pressure on them to be kept. The concept of phylogenetic profiling was first tested on predicting functional relationships between *E. coli* proteins based on their phylogenetic profile across 16 fully sequenced organisms including *S. cerevisiae, B. subtilis*, and *H. influenza* (Pellegrini et al., 1999). Aside from grouping functionally diverse genes to common processes, phylogenetic profiling also offers a route toward providing annotation for otherwise uncharacterized sequences. Even without any knowledge of function of a particular gene, knowing that it is functionally linked to other genes already provides valuable information and entry points for further functional characterization.

Following the pioneering work of Pellegrini and co-workers, the basic concept of phylogenetic profiling has found many applications, e.g., to predict protein-protein interactions (Pagel et al., 2004; Kim and Subramaniam, 2006) or to identify specific enzymes involved in the biosynthesis of particular metabolites in fungi (Ternes et al., 2006). Since its inception, the methodological foundation of phylogenetic profiling has been refined by testing the suitability of orthologous vs. paralogous gene relationships (Skunca et al., 2013), by utilizing structural information for improved homology assignments (Ranea et al., 2007), by implementing novel distance metrics for the measurement of profile similarity (Vert, 2002), as well as by estimating the required number of species for successful phylogenetic profiling applications (Škunca and Dessimoz, 2015). Recently, ProtPhylo, a convenient web-based services for the search for proteins that are possibly associated with a reference protein according to phylogenetic profiling has been developed (Cheng and Perocchi, 2015). ProtPhylo also allows establishing links between species-specific phenotypes and associated candidate proteins.

In this study, we tested the applicability of phylogenetic profiling specifically to the plant metabolic pathway assignment problem. We were especially interested in functional assignments of plant-specific secondary metabolism pathway genes and to gauge the accuracy of phylogenetic profiling given the currently available plant genome sequence and annotation information. Plant secondary metabolites are of particular economic and medicinal interest as many of them have properties proving beneficial in nutrition and medical applications (Singh and Bhat, 2003; Schmidt et al., 2007). To efficiently and reliably assess a plant's secondary metabolite inventory bears tremendous economic potential and provides the basis for targeted pathway engineering (Verpoorte and Memelink, 2002; Oksman-Caldentey and Inze, 2004). Unlike primary metabolism pathways, secondary metabolism pathways often function as independent units with low levels of functional dependencies and, for the rationale of phylogenetic profiling more importantly, impact on other biochemical functions (Hartmann, 1996; Higashi and Saito, 2013). Thus, the set of genes associated with a particular secondary pathway may emerge and disappear independently of other pathways. And as specific metabolic pathways occur only in a subset of species (Pichersky and Gang, 2000), the phylogenetic profiling approach should be ideally suited to identify secondary metabolite pathways from the presence-absence phylogenetic profile of their enzymes. Furthermore, with the massive increase of available whole genome data, the necessary data basis may now be available to put phylogenetic profiling to the test and into practice.

We implemented a comprehensive and rigorous testing scheme covering 39 plant species and 40,960 functionally characterized enzyme genes. While proving successful when tested on specific pathways, a demonstration of the general suitability of phylogenetic profiling is presently severely hampered by the paucity of secondary pathways occurring only in a subset of species. Many pathways are annotated to occur in nearly all plant species, and hence, the very basis of phylogenetic profiling—presence in only a subset of species—is often not fulfilled rendering demonstrating the true potential of phylogenetic profiling challenging. Furthermore, we demonstrate that our abstraction of pathways into isolated units critically impacts the applicability of phylogenetic profiling. Nonetheless, we believe this study to provide a valuable systematic feasibility test highlighting the needs for continued experimental annotation work, while at the same time, demonstrating that phylogenetic profile holds tremendous promise to fill the gaps in our knowledge of plant metabolism.

## Materials and methods

Phylogenetic profiling operates by assigning a particular gene-encoded molecular (here enzymatic) function as present or absent in a given species. Genes with similar presence-absence profiles across several species are then presumed to be involved in the same functional process, in our case, metabolic pathway. The presence/absence call is based on the notion that sequence-similar genes perform the same function. Consequently, the threshold of acceptable sequence-similarity level to assume identical function needs to be decided upon. Clustering all genes encoded in a given set of species based on their sequence homology relationships results in sets of genes with an assumed identical function. The species memberships of every cluster member will define the phylogenetic profile of a given cluster. Here, we refer to such clusters as gene families, or more generally, gene objects. Gene families/objects can also consist of one gene member as well, which will be denoted as singletons. Following the rationale of phylogenetic profiling, gene families should (i) encode one and only metabolic function, (ii) different gene families encode different functions, and (iii) gene families with identical phylogenetic profile should be involved in the same metabolic function.

Pursuing this logic, the following processing steps and approaches to testing its validity were implemented. (A) Based on information available in the database Ensembl Plants (Kersey et al., 2016) and additional filtering steps, gene families were created for the complete known gene inventory of 39 plant species. (B) For every gene family, phylogenetic profiles were generated based on the species origin of all its member sequences. (C) Gene families were tested to correctly reflect a common and unique function and also whether identical phylogenetic profiles of different gene families suggests involvement in a common process; i.e. metabolic pathway. Performance testing of phylogenetic profiling as a means of assigning pathway associations was based on annotation data as available in the data bases Ensembl Plants (Kersey et al., 2016)as well as KEGG (Kanehisa and Goto, 2000). (D) Observed performance results were compared to randomized data to assess statistical significance.

All 39 Ensemble plant species considered in this study along with their KEGG presence annotation are listed in Table 1.

**Table 1:** Plant species and genomes used in this study.

**Ensembl plant species**	**In KEGG**	**Ensemble plant species**	**In KEGG**
Aegilops tauschii (ATA)		Oryza meridionalis (OME)	
Amborella trichopoda (ATR)	Y	Oryza nivara (ONI)	
Arabidopsis lyrata (ALY)	Y	Oryza punctata (OPU)	
Arabidopsis thaliana (ATH)	Y	Oryza rufipogon (ORU)	
Brachypodium distachyon (BDI)	Y	Oryza sativa (DOSA)	Y
Brassica oleracea (BOL)		Ostreococcus lucimarinus (OLU)	Y
Brassica rapa (BRP)	Y	Physcomitrella patens (PPA)	Y
Chlamydomonas reinhardtii (CRE)	Y	Populus trichocarpa (POP)	Y
Cyanidioschyzon merolae (CME)	Y	Prunus persica (PPER)	Y
Glycine max (GMX)	Y	Selaginella moellendorffii (SMO)	Y
Hordeum vulgare (HVU)		Setaria italica (SITA)	Y
Leersia perrieri (LPE)		Solanum lycopersicum (SLY)	Y
Medicago truncatula (MTR)	Y	Solanum tuberosum (SOT)	Y
Musa acuminata (MAC)	Y	Sorghum bicolor (SBI)	Y
Oryza barthii (OBA)		Theobroma cacao (TCC)	Y
Oryza brachyantha (OBR)	Y	Triticum aestivum (TAE)	
Oryza glaberrima (OGL)		Triticum urartu (TUR)	
Oryza glumaepatula (OGU)		Vitis vinifera (VVI)	Y
Oryza indica (OIN)		Zea mays (ZMA)	Y
Oryza longistaminata (OLO)			

*List of all 39 plant species and their abbreviations available in Ensembl Plants and their presence in KEGG (24 plant species)*.

### Sequence and homology information

As we were interested in enzymatic activities, all genes, their sequences, functions, and their pairwise similarity measures were considered based upon their respective protein sequences. Homology information and protein sequences of metabolic pathway enzymes for the 39 plant species (Table 1) available in the Plant Mart database were downloaded from Ensembl Plants (Kersey et al., 2016). The Biomart tool was employed for species selection and retrieving all paralogous and orthologous genes, their sequence identity, homology confidence assignments, and their EC numbers (Kinsella et al., 2011).

### Clustering of genes into gene families

All genes were clustered according to their sequence-based homology to assign genes and their performed functions as either present or absent in a given species. The set of mutually homologous sequences forms a set of genes that, in effect, can be considered a single object consisting of either multiple sequences referred as a gene family or a single sequence in cases where no homologous sequence was found. The latter is being referred to as singletons. Every such object (gene family or singleton) will then be assigned a phylogenetic profile reflecting its presence or absence across all considered plant species along with the respectively performed enzymatic function. We operate under the assumption that all sequences clustered together into a gene family perform the same enzymatic function.

The Ensembl Plants database provides a list of orthologous and paralogous genes as well as pairwise sequence identity values for all included genes. Additionally, an orthology confidence value (low or high) obtained by comparison with the phylogenetic tree is provided (Vilella et al., 2009). In this study, only high-confidence orthology relationships were used.

Pairwise homology relationships between all genes from all 39 species were filtered with regard to percent sequence identity relative to the shorter of two compared sequences as reported in Ensembl imposing two different thresholds of 30% and 70% sequence identity (protein alignments), respectively. All genes not belonging to any multi-member gene family were considered singleton genes. Combining both, the Ensembl-reported homology relationship and the sequence identity, a network was created with genes representing its nodes that are connected if reported homologous and passing the set sequence identity threshold. Connected components of this gene network as detected using the R package *igraph* (Csardi and Nepusz, 2006) are considered gene families. A connected component is defined as a subgraph, in which all nodes are connected, i.e., there exists a path between all nodes of the subgraph. In the above procedure, paralogous and orthologous gene relationships were treated equally. The two clusterings of genes into gene families based on 30% or 70% sequence identity networks will be referred to as Network30 and Network70, respectively.

### Phylogenetic profiles

Phylogenetic profiles were created for each gene object including gene families and singleton gene. Gene objects encoding a particular function were considered present in a particular species if at least one of its member genes was found present in it, otherwise the gene object was considered absent. The presence/absence call across all 39 considered plant species then represents a gene object's phylogenetic profile encoded by ones (indicating presence) and zeroes (indicating absence). Gene objects with identical phylogenetic profiles were then grouped together. As we assumed each gene object to be associated with one unique enzymatic function, gene objects clustered together by identical phylogenetic profiles are then assumed to be involved in the same enzymatic process (pathway). The validity of this statement is the focus of this study and tested by the following statistical procedure.

### Enzymatic pathway information

Metabolic pathway and functional annotation data for all species shared by KEGG and Ensembl Plants were downloaded from the KEGG database (Kanehisa and Goto, 2000). Functional annotation obtained from the KEGG database was assigned to all genes and their respectively encoded proteins. For all plant secondary and primary metabolite pathways contained in KEGG, pathway map numbers referring to the actual biochemical pathway are provided. In total, 40,960 metabolic enzyme genes from 24 plant species with available EC-number from Ensembl Plants were available for pathway analysis.

Two levels of metabolic gene pathway assignments were tested: metabolic classes and metabolic pathways. Metabolic classes were taken as assigned by the KEGG database and include the 10 primary and secondary metabolic pathway classes amino acid metabolism, biosynthesis of other secondary metabolites, carbohydrate metabolism, energy metabolism, glycan biosynthesis and metabolism, lipid metabolism, metabolism of cofactors and vitamins, metabolism of other amino acids, metabolism of terpenoids and polyketides, and nucleotide metabolism. A more detailed classification of enzyme genes was used by considering 94 actual pathway maps associated with the 10 pathway classes as available from KEGG and which are classified as “metabolism” and which have non-zero counts of assigned plant genes (see Supplementary Table 1). Genes were counted toward secondary metabolism pathways only if annotated to only participate in secondary metabolism pathways. Genes annotated to both primary and secondary metabolism pathways were considered primary metabolism genes. The pathway class “Overview” and the associated four detailed pathway maps were not considered as they can be considered unspecific and were not contained in the Ensembl plants data either. Pathways assigned to the classes “metabolism of terpenoids and polyketides“ or “biosynthesis of other secondary metabolites” were considered secondary metabolism pathways. In total, 31 KEGG maps of which 17 carried plant gene annotations were considered as secondary, all others as primary metabolic pathways.

### Evaluation of gene family assignment

The adjusted Rand index (Hubert and Arabie, 1985) was applied to evaluate the validity of gene family assignment in comparison to the known metabolic functions of genes. The *adjustedRandIndex* function of the R package *mclust* (Fraley and Raftery, 1999) was used to compare the clustering of genes into gene families suggesting identical function to those clusterings based on EC number annotation reflecting true function. The resulting Rand index evaluates the degree of accordance of both classifications with zero indicating random, and a value of one signifying perfect agreement. Multiple/ambiguous EC number annotations were treated as distinct true functional annotations such that two genes were only then considered to perform the same function, if both had the same set of EC numbers.

### Phylogenetic profile comparison statistic

#### Test for enrichment of identical profiles within distinct pathways

For each of the 10 metabolic classes and 94 metabolic pathways, the respectively annotated enzyme genes were obtained. Note that genes can come from any of the 24 plant species annotated in KEGG and still map to the same metabolic class or pathway. For each resulting set of n_e_ enzyme genes associated with one particular metabolic class or pathway, the associated set of n_f_ gene objects (gene families and singletons) was determined by identifying the gene object to which the genes were assigned based on the procedure explained above. Each gene object is associated with exactly one phylogenetic profile, P. First it is determined, what fraction, F_pw_, of the n_f_ phylogenetic profiles is identical among all possible comparisons between all n_f_ gene objects belonging to a metabolic class or pathway computed as:

(1) $$\begin{array}{r} F_{pw}=\frac{\sum_{i}^{n_{f}}\sum_{j=i+1}^{n_{f}}\delta_{P_{i},P_{j}}}{\frac{n_{f}\;\left(n_{f}-1\right)}{2}}, \end{array}$$

where δ is the Kronecker delta function yielding 1 in case of identical phylogenetic profiles P_i_ and P_j_. Profiles were considered identical if they had exactly the same bit vector indicating presence and absence across all 39 considered plant species. Likewise, the fraction, F_all_, of identical profiles among all n_f_all_ gene objects associated with all enzyme genes regardless of metabolic class or pathway assignment with n_f_all_ = n_f_ + n_allO_, where n_allO_ is the number of gene objects not assigned to the pathway class or pathway under testing, computes as:

(2) $$\begin{array}{r} F_{all}=\frac{\sum_{i}^{n_{f}}\sum_{j=i+1}^{n_{f}}\delta_{P_{i},P_{j}}\;+\;\sum_{i=1}^{n_{f}}\sum_{j=1,}^{n_{f_{allO}}}\delta_{P_{i},P_{j}}}{{\frac{n_{f\;}\left(n_{f}-1\right)}{2}\;+\;n}_{f}*n_{f_{allO}}}. \end{array}$$

Note that comparisons or phylogenetic profiles in Equation (2) are performed for only those profiles that are part of a particular metabolic class or pathway. The ratio of F_pw_ to F_all_ yields an enrichment, E = F_pw_/F_all_, of identical profiles within a set of pathway gene objects relative to all gene families in the data set. Note that in Equation 2, two set comparisons are combined appearing as summands in the denominator and delimiter, respectively: the within class/pathway profile comparison and the comparison to all other outside-profiles.

Empirical *p*-values of the enrichment score were computed by randomly drawing the same number of gene families as originally annotated to belong to a particular metabolic class or pathway from all gene objects and performing computing enrichment scores for 10,000 such random runs resulting in average random values of F_pw_, F_all_, and an associated enrichment, E_r_. An empirical *p*-value was computed denoting the fraction of equal or larger enrichment scores obtained in the 10,000 random trials than for the actual pathway gene objects set. The obtained *p*-values were corrected for multiple testing—as many metabolic classes or pathways were tested—by using the Benjamini-Hochberg correction implemented in the *p.adjust* function of R.

#### Test for predictability of pathway association based on phylogenetic profiles

For testing the predictive power of phylogenetic profile similarity of two gene objects with regard to their pathway association, the following two procedures were implemented. First, we tested whether increased phylogenetic profile similarity between to genes leads to an increased chance of both genes participating in the same enzymatic pathway. Secondly, we pursued a machine learning approach testing whether pathway membership for a given single gene can be predicted directly from its phylogenetic profiles alone. In greater detail, in the first approach, repeated 100,000 times, two gene objects, g_1_ and g_2_, was selected at random from the set of all 2,206 including gene families and singletons. The phylogenetic profiles PP_1_ and PP_2_, each a 39-element vector consisting of ones (presence in a species) and zeroes (absence a species), associated with g_1_ and g_2_, respectively, were compared by their Jaccard index measuring the intersection vs. the union of “1” entries and their distance, d_PP_, defined as:

(3) $$\begin{array}{r} d_{PP}=\frac{|PP_{1}\&PP_{2}{|}_{1}}{|PP_{1}\;|\;PP_{2}{|}_{1}}, \end{array}$$

where “&” and “|” are the bitwise AND and OR operator, respectively, and ||_1_ is the L1-norm; i.e., the sum of all ones in the PP-vectors of length 39. For both gene objects, g_1_ and g_2_, all KEGG pathway maps to which their member genes are annotated were determined and the agreement, A_PW_g1,g2_, between both pathway lists measured following the Jaccard index logic as:

(4) $$\begin{array}{r} {A\text{\_}PW}_{g1,g2}=\frac{PW_{g1}\cap {PW}_{g2}}{min\;\left(N_{1},{\;N}_{2}\right)}, \end{array}$$

where *PW*_*g*1_ and *PW*_*g*2_ are the lists of pathways associated with gene objects *g*_1_ and *g*_2_, respectively, *N*_1/2_ are the numbers of different pathways in *PW*_*g*1_ and *PW*_*g*2_, the intersection represents the number of pathways found in both pathway lists. Note that we deliberately decided to sample based on gene objects and not based on individual genes as the latter would bias the result to large gene families.

Value pairs of d_PP_ and A_PW_g1,g2_ from all random trials were plotted as a scatter plot (**Figure 7**) and a logistic function A_PW = f (d_PP_) with A_PW = 1/ (1 + exp (−a ^*^ (d_PP_ – b))) was fitted to the data using the non-linear fit function “nls” of R and a and b being parameters to be determined by the fit. The logistic function was chosen as it naturally converges to zero and one, the two possible extreme values of A_PW.

For the machine learning approach, the Clus-HMC software (Schietgat et al., 2010; Skunca et al., 2013) was used to predict the metabolic pathway class or detailed pathway (KEGG map) for every gene object (gene family or singleton gene) based on its phylogenetic profile. The Clus-HMC package is ideally suited as it allows for multi-label objects (a gene object and its function can be assigned to more than one pathway map) and because it deals with hierarchical data (metabolism class and individual KEGG maps as the lower level). Clus-HMC employs decision trees as the classification engine. We used it in Random Forest mode with 50 trees per run, Jaccard-distance metrics, and prediction performance reported on out-of-bag examples; i.e. the internal cross-validation, employed typically as part of the Random forest methodology. Performance was judged by the area under the precision-recall curve (AUCPRC), where precision is defined as the ratio of true positive prediction to the sum of true positive and false positive predictions; i.e., of all predictions made, what fraction is correct. Recall is defined as the ratio of true positive predictions to the sum of true positive and false negative predictions; i.e., of all positive examples in the dataset, what fraction was retrieved as positive predictions. Larger values of the AUCPRC indicate better predictions. Because our data is heavily imbalanced—for any given gene family, only one or few pathways out of all possible will be assigned to them—the better known area under ROC (true positive vs. false positive rate) would be misleading (Davis and Goadrich, 2006). In total, 2,206 gene objects (gene families and singleton genes) associated with 816 unique phylogenetic profiles were tested to be assigned to either 10 metabolic pathway classes or 94 detailed pathway maps. AUCPRC values obtained for true associations of gene objects and their phylogenetic profiles with metabolic pathway classes and maps were compared to AUCPRC values obtained for randomized assignments by randomly redistributing the 10 metabolic pathway classes and 94 pathway maps to all gene objects while preserving their occurrence and avoiding repeated assignments of a gene object to the same pathway class or map. This randomization process was repeated 100 times for pathway class predictions, and 20 times for pathway maps. For the latter, fewer random runs were necessary as their number (94 maps vs. 10 classes) was much larger. Statistical comparisons of true to random predictions were performed using the non-parametric Wilcoxon rank sum test and averaged over all performed repeat randomization runs.

#### Phylogenetic profile similarity as an indicator of gene co-expression and protein-protein interactions

Phylogenetic profile similarities of two gene objects were tested for being informative with regard to co-regulation of their gene expression and physical interactions of their encoded products via protein-protein interactions focusing on *Arabidopsis thaliana* as the reference species given the rich experimental information available for this model plant species with regard to both gene expression and protein-protein interactions. Gene expression information was obtained from NASCArray (Craigon et al., 2004) covering a broad range of experimental condition probed by about five thousand ATH1 Affymetrix gene chip gene expression experiments (hybridizations). Raw gene expression data were log-transformed and quantile-normalized as explained in Korkuc et al. (2014). To reduce computation time, a random subset of samples drawn with 10% chance from the original NASCArray sample set and resulting in expression data for 20,922 genes across 479 hybridizations was used for analysis. For all possible pairs of 500 randomly selected Arabidopsis enzyme genes, their phylogenetic profile similarities, d_PP_, were plotted vs. their pairwise Pearson correlation coefficient, r_GE_, of gene expression across the 479 gene expression samples. In total, for 93,961 Arabidopsis gene pairs, both phylogenetic as well as gene expression information was available for both genes forming the pair of enzymes allowing to test whether increased phylogenetic similarity corresponds to increased correlation of their gene expression.

Physical interactions of Arabidopsis proteins were obtained from the database AtPIN (Brandao et al., 2009). Interactions with experimental support were considered only totaling in 95,219 pairwise protein-protein interactions among 14,995 unique proteins of which 5,978 pairs formed among 2,265 genes were contained in the functional annotation data as well identifying them as enzymes. For all enzyme pairs reported to physically interact, we determined their associated phylogenetic profile similarity, d_PP_, and compared the resulting distribution to the distribution of d_PP_-values associated with enzyme pairs not reported to interact. Statistical significance was established based on the non-parametric Wilcoxon rank sum test.

## Results

**Figure 1:**
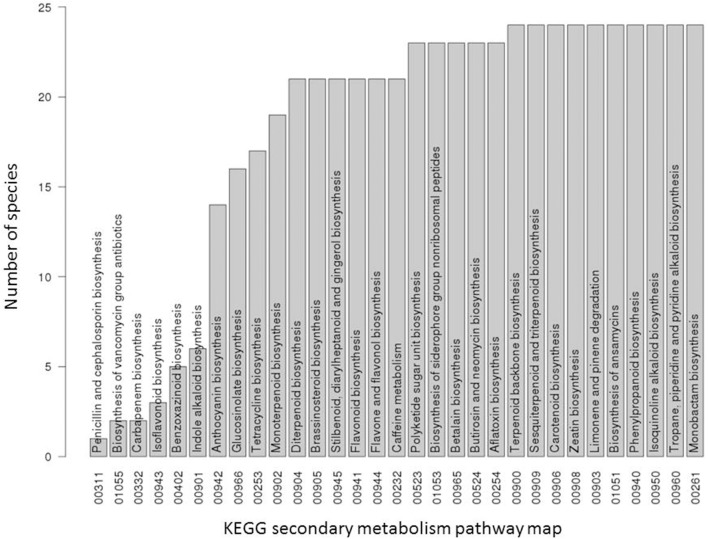
Occurrence statistic of secondary metabolite pathways in 24 KEGG species. For each secondary metabolic pathway of the KEGG database, the number of plant species containing it out of all 24 plant species used in this study are displayed. A pathway was considered present in a given species if at least one enzyme gene from this species was assigned to this pathway.

The rationale of phylogenetic profiling posits that genes collectively performing a particular biological function are present as a set in only those species in which the function is performed. And in order for phylogenetic profiling to be specific, particular functions should be associated with unique phylogenetic profiles. As the goal of this study was to exploit phylogenetic profiling for metabolism pathway assignments of genes with a focus on secondary metabolism, we first inspected the presence of known secondary metabolism pathways across the 24 plant species with available Ensembl and KEGG information (Figure 1). Initially, pathways were considered present in a particular species if at least one gene was found in this species that was annotated to belong to this pathway. Based on this presence/absence call, about one third (10 out of all 31 secondary pathways) were found present in all 24 plant species. Thus, for those pathways, no differential presence/absence profile was evident rendering the application of phylogenetic profiling unspecific as a number of different secondary metabolism pathways exhibit the same presence profile. Evidently, this result reflects the current breadth of species coverage available in KEGG and Ensembl. Seven pathways were detected present in less than half of all KEGG species and another 14 were found in almost, but not, all 24 species (Figure 1). Thus, the seven pathways with confined species coverage appear most promising with regard to verifying phylogenetic profiling as an annotation means provided that their species spectra do not extensively overlap. They include the pathways “penicillin and cephalosporin biosynthesis,” “biosynthesis of vancomycin group antibiotics,” “carbapenem biosynthesis,” “isoflavonoid biosynthesis,” benzoxazinoid biosynthesis,” “indole alkaloid biosynthesis,” and “anthocyanin biosynthesis.” Note that the pathway “penicillin and cephalosporin biosynthesis” appears listed in the plant dataset obtained from KEGG. Both antibiotics are known to be produced in fungi, but not plants. The plant annotation in KEGG originates from an enzyme of the red algae *Cyanidioschyzon merolae* annotated as similar to D-amino acid oxidase, which is known to catalyze a reaction in the penicillin and cephalosporin biosynthesis pathway and was also assigned to primary metabolite pathways of amino acid metabolism. As this pathway is not actually performed in plants, it was not considered further in this study. Likewise, the bacterial vancomycin pathway was not considered further either.

The analysis of the species-per-pathway distribution was also performed for primary metabolite pathway enzymes annotated in the KEGG database. As expected for primary metabolism pathways, because they represent essential functions required for survival, the majority (71 out of 81 primary metabolism pathways annotated in KEGG for the 24 plant species) were found present throughout all species (data not shown).

**Figure 2:**
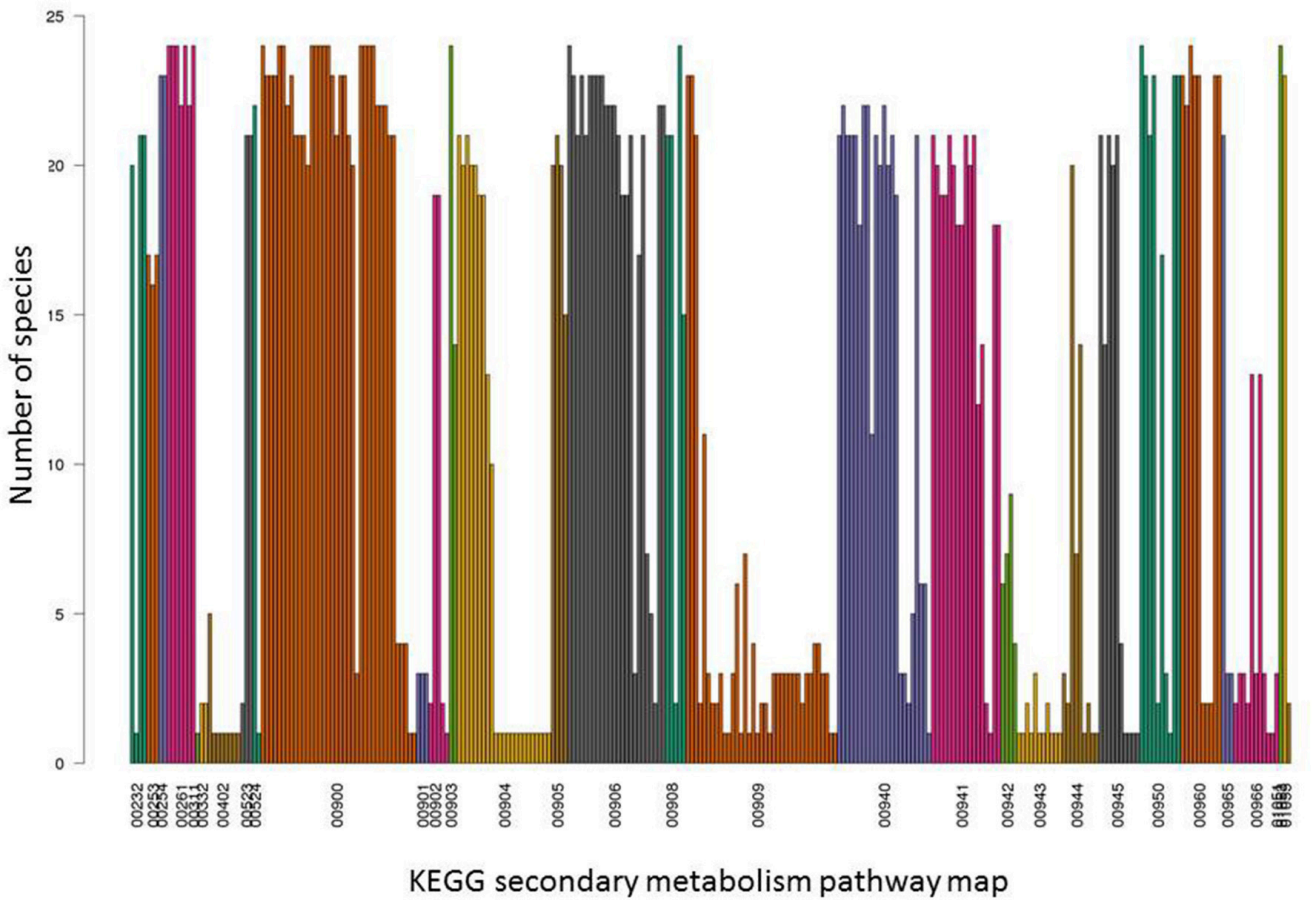
Detailed occurrence profiles of secondary metabolite pathways. For all 31 KEGG secondary metabolite pathways indicated by their KEGG map number, presence across the 24 plant species used in this study is plotted for their respective constituent enzymes. Each bar represents one pathway enzyme and their presence across 24 plant species based on identical EC number annotation in the different species. Enzymes are grouped and colored according to their KEGG map number. For associated pathway names, see Figure 1.

**Figure 3:**
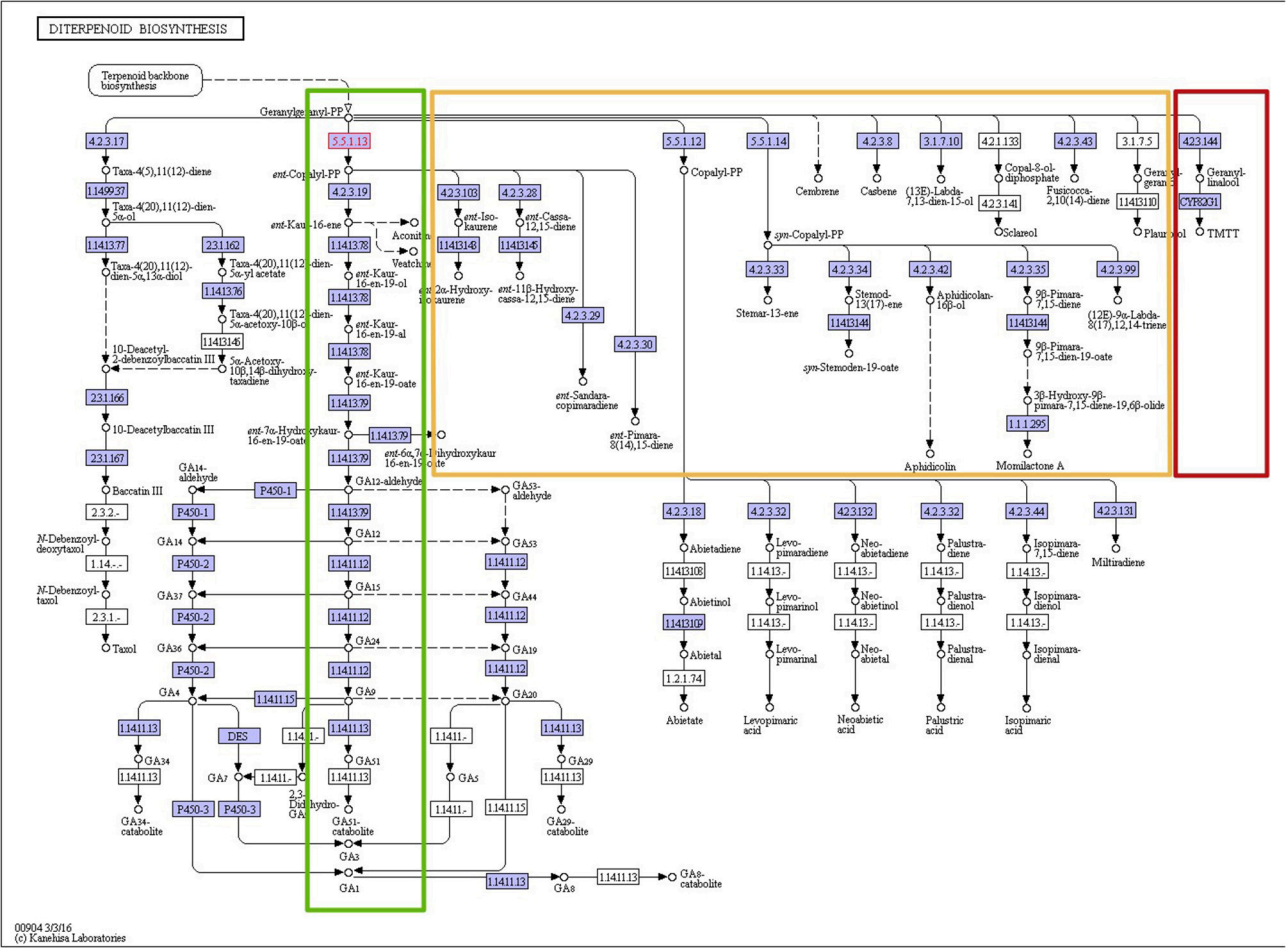
KEGG pathway map of the diterpenoid biosynthesis pathway (map 00904). All enzymes performing the steps of the linear main pathway (highlighted green) were found present in at least 19 plant species. By contrast, the orange-highlighted pathway branches were found present in only one or two species each, and the dark-red highlighted branch was found present in 13 species. Permission to reproduce this pathway map image was kindly granted by the KEGG curators.

In the initial presence/absence profile of secondary metabolite pathways across all 24 plant species, presence was considered confirmed if at least one component gene was annotated present in a given species. As pathways are composed of several enzymes (with an average of 11 enzymes per secondary metabolism pathway based on the dataset used in this study), and furthermore, individual pathways may consist of pathway branches acting semi-independently, a more detailed analysis based on pathway member genes was performed. Indeed, when inspecting the presence of individual pathway member genes, we observed that, while particular member genes of a given pathway were indeed found across all species, other genes associated with the same pathway may very well exhibit a very narrow species presence spectrum (Figures 2, 3). For example, in the pathway “diterpenoid biosynthesis” (KEGG map number 00904) about half of the member genes were found present in the majority of species, while the other half was detected present in few (one) species only (Figure 3). The pathway “sesquiterpenoid and triterpenoid biosynthesis” (KEGG map 00909) is another example of gene-specific presence profiles associated with the same pathways. By contrast, for other pathways, all member genes were detected to occur in essentially all species (e.g., “terpenoid backbone biosynthesis,” KEGG map 00900 or “flavonoid biosynthesis,” KEGG map 00941, or consistently in only few species (e.g., isoflavonoid biosynthesis, KEGG map 00943).

The detailed presence/absence profiles displayed in Figure 2 already reveal a critical limitation of phylogenetic profiling. The notion of collective presence or absence may not always be fulfilled given our abstraction of isolated biochemical pathways and may require further subdivision of distinct biochemical reactions and functions. This is illustrated for the diterpenoid biosynthesis pathway (Figure 3). While the enzymes of the main branch of this pathway were found present in nearly all of the 24 species with available pathway annotation, the pathways branching off of the main path are present in selected species only.

**Figure 4:**
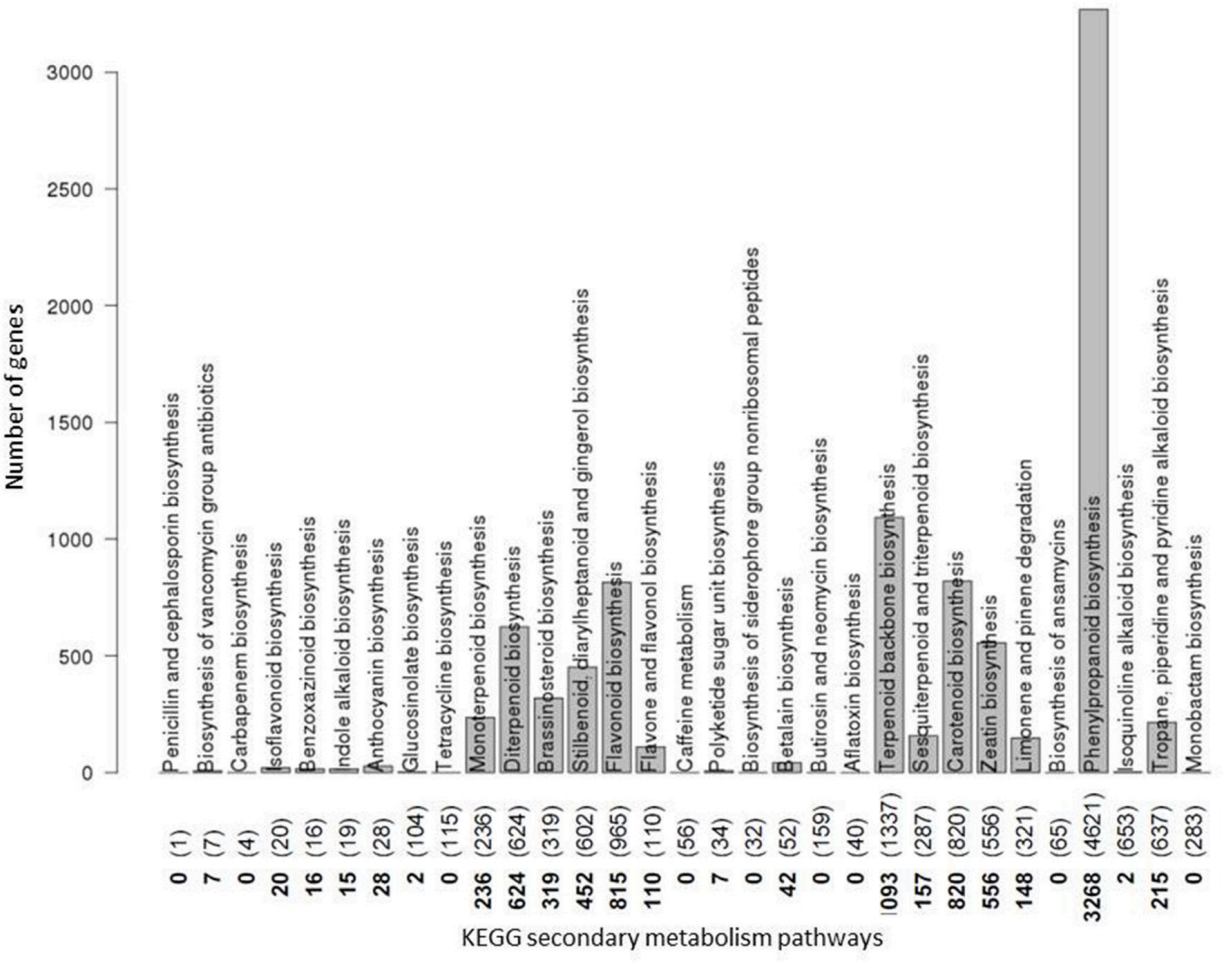
Number of genes coding for enzymes involved in secondary metabolite pathways. The number of genes assigned to each plant secondary metabolite pathway as annotated in the KEGG database is plotted. Counts are based on all plant genes from any of the 24 plant species considered in this study. Numbers along the abscissa denote the actual number of genes involved in secondary metabolism pathways only with numbers in parentheses referring to the respective counts when considering genes annotated to participate in both secondary and primary metabolism pathways. Pathways are indicated by their names. Pathways “Biosynthesis of vancomycin group antibiotics” as a bacterial pathway, and “Penicillin and cephalosporin biosynthesis” as a fungal pathway were not considered any further in this plant-focused study. For the pathways “Benzoxazinoid biosynthesis” and “Isoquinoline alkaloid biosynthesis,” while listed in KEGG, conflicting or no annotations were contained in Biomart and, therefore, were discarded from further analysis. All remaining 19 secondary metabolism pathways with non-zero KEGG gene counts were considered further.

The number of annotated genes involved in a pathway proved greatly variable (Figure 4). Considering as associated genes only those that are annotated to exclusively participate in secondary and not primary metabolite pathways, not all of the 31 secondary metabolism pathways actually contain gene assignments, with nine pathways without any secondary metabolite pathway specific genes assigned to them (e.g. “caffeine metabolism”), and others contain only very few (e.g. “anthocyanin biosynthesis”). By contrast, 13 pathways have hundreds (“flavonoid biosynthesis, “carotenoid biosynthesis,” “diterpenoid synthesis”) and even thousands (“phenylpropanoid biosynthesis,” “terpenoid backbone biosynthesis”) of genes annotated to them. Note that this gene count includes all orthologues and paralogs across all 24 plant species considered here and is based on EC number annotation as provided by KEGG.

Summarizing these initial survey data, it is apparent that, despite the large volumes of accumulated sequencing and genome annotation data, actual secondary pathway knowledge with regard to species and gene coverage as well as suitability for testing phylogenetic profiling approaches is relatively limited and confined to only few secondary metabolite pathways.

### Evaluation of gene family assignment

Assigning homology amongst all genes of all considered plant species is a crucial step in phylogenetic profiling as its outcome directly defines the presence-absence profile of particular enzymatic activities across the considered plant species.

We operated under the assumption that all members of a gene family perform one and one function only, and that different gene families perform different functions. To test this assumption, we compared the assignments of genes to gene families to the functional assignments as given by the KEGG EC number, with a total of 994 different KEGG enzyme identifiers in the dataset indicating that many different enzymatic activities. Our network-based approach with a sequence identity threshold of 30 percent (Network30) resulted in 2,206 gene objects including 1,686 gene families and 520 singleton genes leading to an adjusted Rand index of 0.471. Using a threshold of 70 percent sequence identity yielded 9,285 objects (4,373 gene families and 4,912 singleton genes), and an adjusted Rand index of 0.116. Thus, the more generous clustering, allowing sequences of greater divergence to be clustered together, yielded a better agreement with actual biological function assignments. By contrast, the partitioning of genes into gene families at higher sequence identity threshold levels seems to under-cluster genes compared to actual function. It must be cautioned, however, that KEGG functional assignments may in turn be based on sequence comparisons. Thus, the two clusterings may not be entirely independent.

As the Network30 gene family assignments proved more consistent with actual biochemical functional annotation, we used it henceforth for testing the phylogenetic profiling methodology to assign pathway relationships.

**Figure 5:**
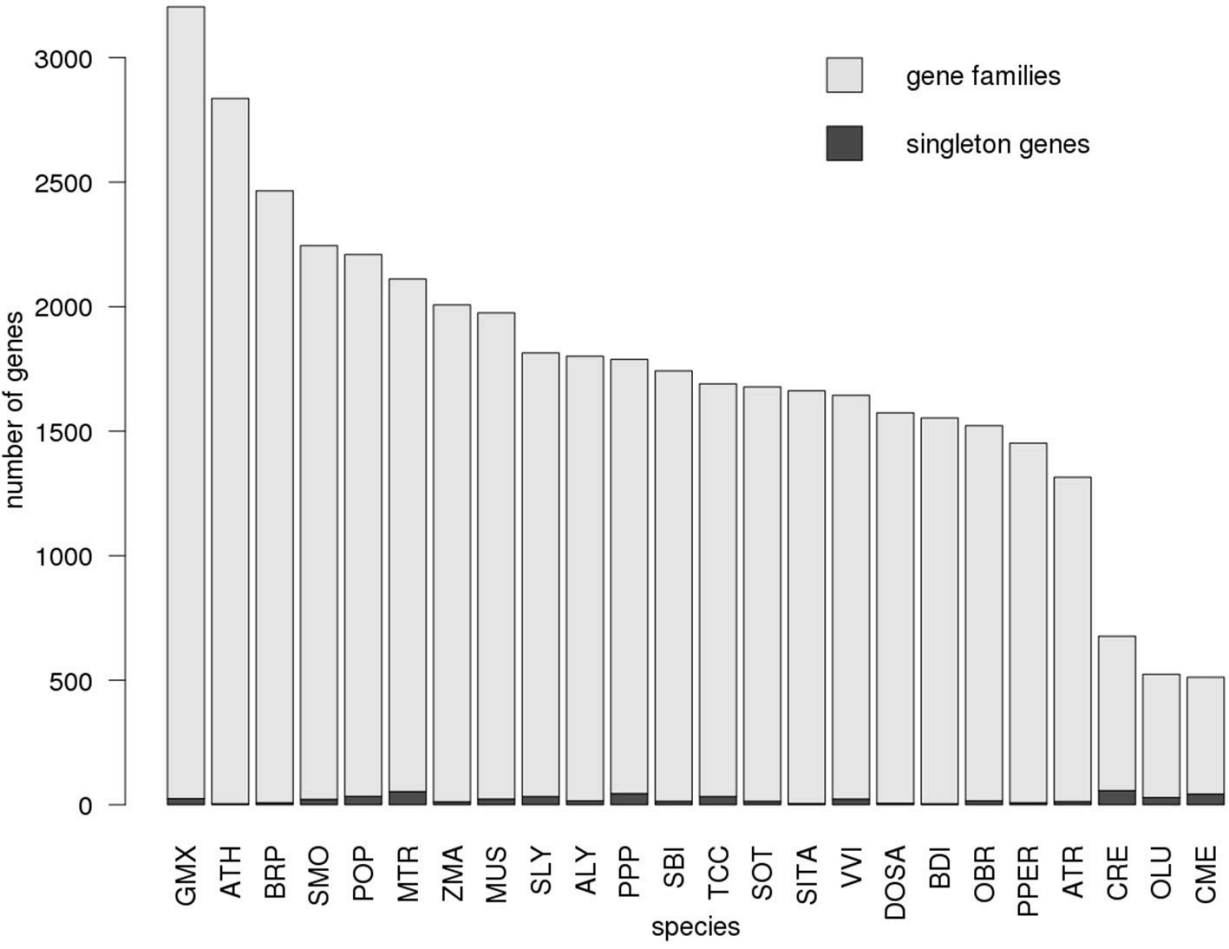
Number of enzyme genes assigned to gene families. The proportion of genes assigned to gene families (light gray) as well as singleton genes (dark gray) is displayed for each plant species with available KEGG and gene annotation. The distribution is shown for gene family assignment based on the Network30 dataset. Species are sorted by the total number of genes. For every species, only genes annotated to function as enzymes were considered.

Figure 5 shows the counts of individual genes annotated as enzymes, associated proportions assigned into gene families and singleton genes, respectively, across all 24 KEGG annotated plant species. Notably, the number of singletons genes does not correlate with the total number of enzymes in the species. Furthermore, the three algal species *Chlamydomonas reinhardtii* (CRE), *Ostreococcus lucimarinus* (OLU), *Cyanidioschyzon merolae* (CME) are characterized by a pronounced lowered number of genes assigned to families, but a proportionally high number of singleton genes likely reflecting their evolutionary distance from higher plants.

### Phylogenetic profiling

#### Enrichment of identical profiles within distinct metabolic pathways

The primary objective of this study was to assess the utility of phylogenetic profiling as a means to associate genes by virtue of identical profiles to a common biological function, here metabolic pathway, and in particular, secondary metabolism pathway. If true that phylogenetic profile agreement implies common pathway involvement, then, for any given known pathway, there should be a higher than randomly expected agreement between phylogenetic profiles associated with genes assigned to it (high values of F_pw_, Equation 1). Because high degree of profile agreement may also simply reflect that the respective profiles occur very frequently across all genes and that are participating in many different pathways, we need to compare the within-pathway agreement relative to the expected agreement based on the general frequency of this phylogenetic profile (F_all_, Equation 2). Note that, when referring to genes, we actually mean gene objects defined as gene families or singleton genes as described above. And every gene object is characterized by a phylogenetic profile. Every actual gene present in a given species is a representative of a gene object, which performs a defined enzymatic function. This assumption is not strictly true (adjusted Rand index of 0.471), but nonetheless forms the operational and reasonable basis of our approach. As outlined above, we based the following analyses on the Network30-based gene family assignments as explained in Methods.

We performed statistical tests considering two levels of metabolic pathway abstraction: the very coarse level of metabolism classes—there are 10 different metabolism classes defined in KEGG (Table 1, note that we did not consider the class “Overview” as being too generic) and the more detailed functional grouping captured as metabolic pathways. In total, we considered 94 different pathway maps with 19 annotated as secondary metabolism. Every pathway belongs to a particular KEGG metabolism class (Supplementary Table 1).

For five of the 10 considered metabolic classes, indeed a significant enrichment (multiple testing adjusted *p*-value < 0.05) of phylogenetic profile agreements of gene objects annotated to the same class relative to random expectation was observed. All five belong to primary metabolism classes and include: “Amino acid metabolism (AAM),” “Metabolism of other amino acids (MOAA),” “Metabolism of cofactors and vitamins (MCV),” “Nucleotide metabolism (NM),” and “Carbohydrate metabolism (CM)” (Table 2). The fold-enrichment levels for the two secondary metabolism classes “Metabolism of terpenoids and polyketides (MTP),” “Biosynthesis of other secondary metabolites (BSM),” while greater than one (1.435 and 1.268, respectively), did not prove to be statistically significant. Of the three remaining metabolic classes, “Energy metabolism (EM)” showed borderline significant enrichment (1.217-fold, adjusted *p*-value = 0.09), while “Lipid metabolism (LM)” and “Glycan biosynthesis and metabolism (GBM)” showed no discernable enrichment (Table 2).

**Table 2:** Statistics of metabolism class assignments and phylogenetic profile identity of gene families and singletons.

**KEGG metabolism class**	**N_GF/S_**	**F_pw_**	**F_all_**	**E = F_pw_/F_all_**	**Adjusted *p*-value**
Amino acid metabolism (AAM)	358	0.037	0.020	1.810	<0.001
Metabolism of other amino acids (MOAA)	114	0.039	0.022	1.787	<0.001
Metabolism of cofactors and vitamins (MCV)	295	0.029	0.019	1.549	<0.001
Nucleotide metabolism (NM)	410	0.021	0.015	1.391	0.008
Carbohydrate metabolism (CM)	742	0.018	0.016	1.130	0.048
Energy metabolism (EM)	261	0.019	0.016	1.217	0.088
Metabolism of terpenoids and polyketides (MTP)	84	0.018	0.013	1.435	0.327
Biosynthesis of other secondary metabolites (BSM)	97	0.012	0.009	1.268	0.809
Lipid metabolism (LM)	271	0.014	0.014	0.981	0.809
Glycan biosynthesis and metabolism (GBM))	145	0.008	0.009	0.930	0.991

*Table lists for all 10 KEGG metabolism classes (KEGG class “Overview” was not considered) the number of gene families or singleton genes (jointly referred to as gene objects, N_GF/S_) annotated to them, the fraction of all profile-profile comparisons among all N_GF/S_ gene objects yielding identical profiles within a class (F_pw_, Equation 1), the fraction of all profile-profile comparisons of N_GF/S_ gene objects yielding identical profiles within and to gene objects outside a class (F_all_, Equation 2), the resulting fold enrichment (E = F_pw_/F_all_) of identical phylogenetic profiles within a class relative to expectation, and 30 associated Benjamini-Hochberg corrected empirical p-value based on 10,000 random class assignments. Results are based on Network30-based gene family assignments (see Methods). Metabolism classes are sorted in ascending order of p-value. Highlighted bold are the secondary metabolism classes*.

**Figure 6:**
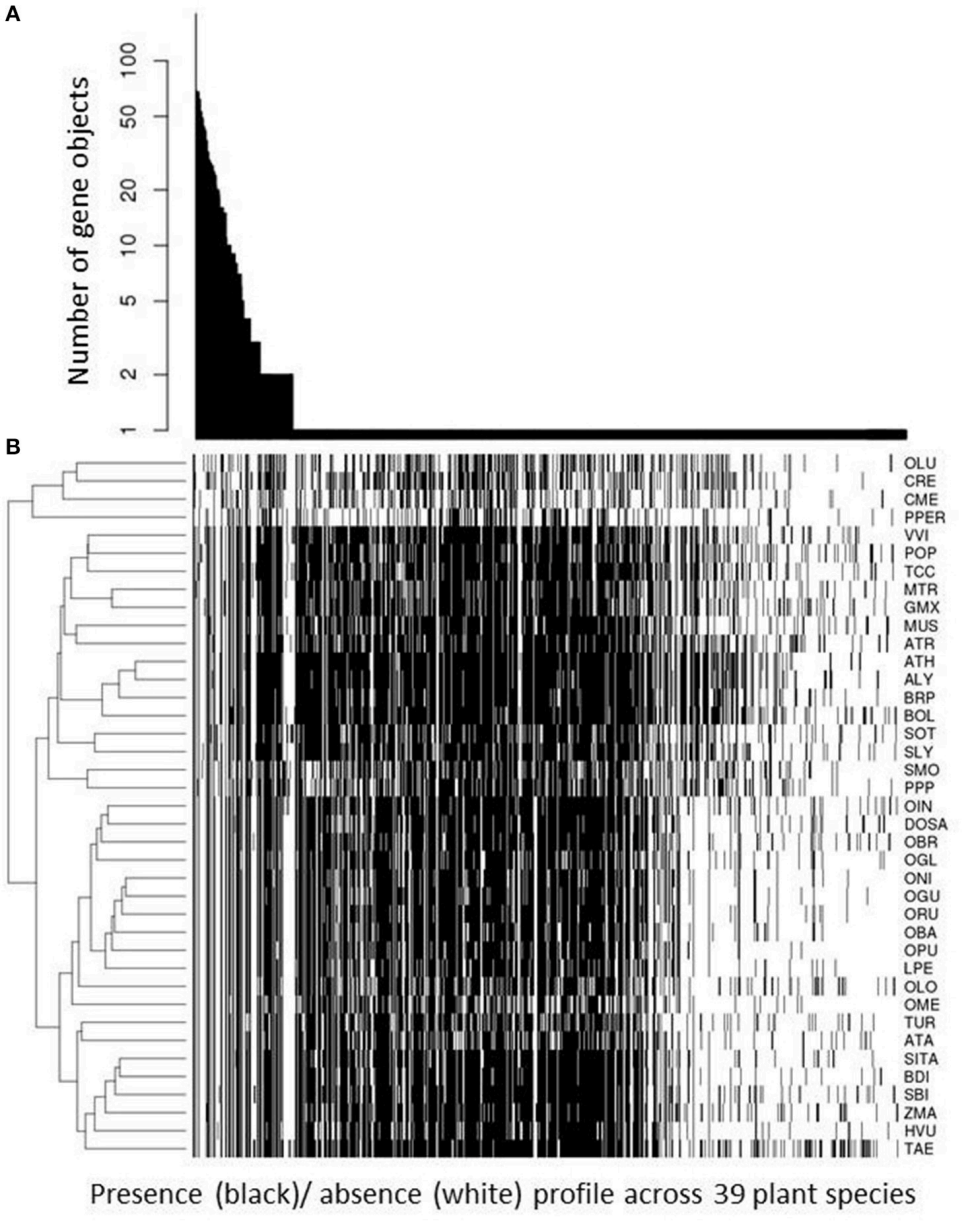
Phylogenetic profile frequency statistic. For all 818 unique phylogenetic profiles, their respective occurrence; i.e. number of gene objects with this profile, is plotted in descending order of occurrence **(A)**. The associated actual presence (black cell)/ absence (white cell) for all 39 plant species considered in this study is plotted underneath **(B)**. Plant species are given by their abbreviation introduced in Table 1 and clustered hierarchically (complete linkage, Euclidean distance) according to similarity of presence/absence pattern. Unique profiles found for only one gene object are sorted in descending number of genes clustered into this object; i.e., from large gene families (grouped to the left) to small gene families and singleton genes (grouped to the right). Note that we used only those gene objects for which a KEGG annotation for at least one member plant was available.

The result of significant phylogenetic profile agreement associated with primary, and therefore, ubiquitous pathways appears surprising at first, given that we argued that phylogenetic profiling is ideally suited to identify biochemical functions confined to subsets of species. However, the rationale still holds and is meaningful. Phylogenetic profiles reflecting presence in all species are informative in the sense that they identify functions that are indispensable, and as many profiles will reflect presence in subsets of species only, even those profiles suggesting presence in all species can be enriched relative to random expectation. And they are enriched exactly in those pathway classes that are indispensable. However, the specificity of pathway assignments may be lost as many different pathways will essential and therefore performed in all species (see Figure 6 and associated results below).

At the more detailed level of functional abstraction considering actual metabolic pathways as annotated in KEGG, 29 of the 94 considered pathways displayed significant enrichments (multiple testing adjusted *p*-value < 0.05) of identical phylogenetic profiles between member gene objects relative to random expectation (Table 3). Again, and following the same rationale as explained above for metabolic classes, most of the 29 pathways belong to primary metabolism pathways such as the TCA-cycle (fold enrichment, *E* = 4.145, adjusted *p*-value < 0.001) or various amino acid metabolism pathways (Table 3). However, the largest and also significant enrichment factors were observed for the two secondary metabolism pathways “Stilbenoid, diarylheptanoid and gingerol biosynthesis” (*E* = 12.553, *p* = 0.024) and the borderline significant pathways “Diterpenoid biosynthesis” (*E* = 11.626, *p* = 0.069) and “Flavonoid biosynthesis” (*E* = 4.695, *p* = 0.097). Of the remaining 17 secondary metabolism pathways, 11 contained three or fewer different gene objects (Supplementary Table 2) rendering any meaningful statistical assessment impossible. Note that we only considered gene objects to be assigned to secondary metabolism pathways that are not also participating primary metabolism processes. Hence, the number of gene objects may be considerably smaller than the number of enzymes annotated in KEGG to belong to a particular secondary metabolism pathway. The secondary metabolism pathway “Limonene and pinene degradation” shows a high enrichment of profile agreements (*E* = 7.296), but statistical significance could not be established (*p* = 0.142). All other secondary metabolism pathways (“Terpenoid backbone biosynthesis,” “Carotenoid biosynthesis,” “Phenylpropanoid biosynthesis,” “Zeatin biosynthesis”) showed no discernable enrichment of profile agreements within them despite relatively high numbers of gene objects (>10) assigned to them (Supplementary Table 2).

**Table 3:** Statistics of metabolism pathway assignments and phylogenetic profile identities of gene families and singletons.

**Pathway map number and name**	**Class**	**N_GF/S_**	**F_pw_**	**F_all_**	**E = F_pw_/F_all_**	**Adjusted *p*-value**
00450 Selenocompound metabolism	MOAA	15	0.152	0.037	4.165	<0.001
00020 Citrate cycle (TCA cycle)	CM	31	0.146	0.035	4.145	<0.001
00920 Sulfur metabolism	EM	23	0.142	0.035	4.014	<0.001
00660 C5-Branched dibasic acid metabolism	CM	15	0.152	0.039	3.958	<0.001
00280 Valine. leucine and isoleucine degradation	AAM	26	0.120	0.036	3.377	<0.001
00710 Carbon fixation in photosynthetic organisms	EM	70	0.065	0.026	2.498	<0.001
00250 Alanine. aspartate and glutamate metabolism	AAM	43	0.063	0.026	2.467	<0.001
00400 Phenylalanine. tyrosine and tryptophan biosynthesis	AAM	49	0.061	0.028	2.188	<0.001
00010 Glycolysis/Gluconeogenesis	CM	105	0.050	0.024	2.134	<0.001
00260 Glycine. serine and threonine metabolism	AAM	75	0.049	0.024	2.068	<0.001
00620 Pyruvate metabolism	CM	72	0.047	0.024	2.000	<0.001
00240 Pyrimidine metabolism	NM	325	0.023	0.014	1.670	<0.001
00300 Lysine biosynthesis	AAM	16	0.125	0.034	3.682	0.006
00650 Butanoate metabolism	CM	25	0.097	0.035	2.746	0.006
00640 Propanoate metabolism	CM	41	0.070	0.029	2.428	0.006
00270 Cysteine and methionine metabolism	AAM	70	0.053	0.025	2.180	0.006
00480 Glutathione metabolism	MOAA	42	0.050	0.023	2.152	0.010
00030 Pentose phosphate pathway	CM	63	0.040	0.024	1.684	0.010
00071 Fatty acid degradation	LM	15	0.114	0.034	3.365	0.015
00945 Stilbenoid. diarylheptanoid and gingerol biosynthesis	**BSM**	12	0.152	0.012	12.553	0.024
00220 Arginine biosynthesis	AAM	41	0.041	0.022	1.865	0.030
00290 Valine. leucine and isoleucine biosynthesis	AAM	35	0.047	0.026	1.820	0.030
00630 Glyoxylate and dicarboxylate metabolism	CM	74	0.035	0.022	1.613	0.033
00051 Fructose and mannose metabolism	CM	65	0.034	0.021	1.616	0.035
00860 Porphyrin and chlorophyll metabolism	MCV	63	0.034	0.021	1.638	0.041
00410 beta-Alanine metabolism	MOAA	24	0.047	0.027	1.740	0.047
00230 Purine metabolism	NM	367	0.021	0.014	1.487	0.049
00310 Lysine degradation	AAM	13	0.077	0.037	2.060	0.050
00760 Nicotinate and nicotinamide metabolism	MCV	22	0.048	0.022	2.214	0.052
00904 Diterpenoid biosynthesis	**MTP**	7	0.143	0.012	11.626	0.069
00380 Tryptophan metabolism	AAM	35	0.039	0.022	1.730	0.091
00941 Flavonoid biosynthesis	**BSM**	14	0.066	0.014	4.695	0.097
00790 Folate biosynthesis	MCV	30	0.039	0.020	2.003	0.097

*For all KEGG metabolism pathways given by their KEGG map number and name and with profiling results corresponding to a False Discovery Rate (FDR) of less than 10%, table lists the metabolism class to which pathway belongs indicated by their abbreviations introduced in Table 1, the number of gene families or singleton genes (referred to as gene objects, N_GF/S_) annotated to them, the fraction of all profile-profile comparisons among all N_GF/S_ gene objects yielding identical profiles within a pathway (F_pw_, Equation 1), the fraction of all profile-profile comparisons of N_GF/S_ gene objects yielding identical profiles within and to gene objects outside a pathway (F_all_, Equation 2), the resulting fold enrichment (E = F_pw_/F_all_) of identical phylogenetic profiles within a pathway relative to expectation, and associated Benjamini-Hochberg corrected empirical p-value based on 10,000 random pathway assignments. Results are based on Network30-based gene family assignments (see Methods). Only pathways with significant enrichment of identical profiles (adjusted p-value < 0.1) are listed. For a complete listing of statistical results for all 94 pathways considered in this study, see Supplementary Table 1. Pathways belonging to secondary metabolism classes Biosynthesis of other secondary metabolites (BSM) and Metabolism of terpenoids and polyketides (MTP) are highlighted using bold face font. Pathways are sorted in ascending order of p-value*.

We considered as potentially functionally linked only those gene objects that share the same phylogenetic profile. While more relaxed thresholds (tolerating a small number of presence/absence mismatches across the 39 considered plant species) or even gradual profile-profile distances based on bit-distances are conceivable, given the all-or-nothing criterion employed here, inspecting the frequency distribution of unique phylogenetic profile may shed further light on the representation of unique profiles across all gene families (Figure 6). In total, we determined 818 unique profiles associated with the 2,206 gene objects (gene families or singleton genes). The present-in-all-species profile was observed most frequently. One hundred and Eighty gene objects were detected with this profile. One hundred and thirty one profiles were observed to be shared by two or more gene objects, while 685 were found uniquely associated with only one gene object (Figure 6). Unique profiles are both those that are characterized by a presence in only a small number of species as well as general presence with unique absences in particular species. Figure 6 also visualizes the presence/absence profiles across the 39 plant species considered in this study. Because it is the most broadly and intensively investigated model plant, *A. thaliana* stands out as possessing the largest number of presence calls. As also the genome annotation of other species is often derived from Arabidopsis based on sequence comparison, other species can essentially only possess fewer, but not more functionally annotated gene objects unless investigated more closely and experimentally or based on de-novo bioinformatic gene annotation. The clustering of plant species according to similarity of their presence/absence profile across all 818 unique profiles reproduces the established phylogenetic relationships between them (Figure 6).

#### Predictability of metabolic pathway association based on phylogenetic profile similarity

We observed that for selected pathway classes (Table 2) and detailed pathways (Table 3), indeed a significant increase of occurrence of genes with the same phylogenetic profile is evident. We now asked, whether the reverse, high profile similarity implies association to the same pathway, holds true as well. While the former can be regarded the necessary condition for the phylogenetic profiles to be of predictive value, the latter constitutes the ultimate test and defines the applicability of phylogenetic profiling in practice. Reversing the viewpoint (pathway association suggests profile similarity vs. profile similarity predicts pathway association) is not equivalent either, because of the general absence of symmetry of conditional probability of two events A and B with P (A|B) ≠P (B|A) in most cases.

**Figure 7:**
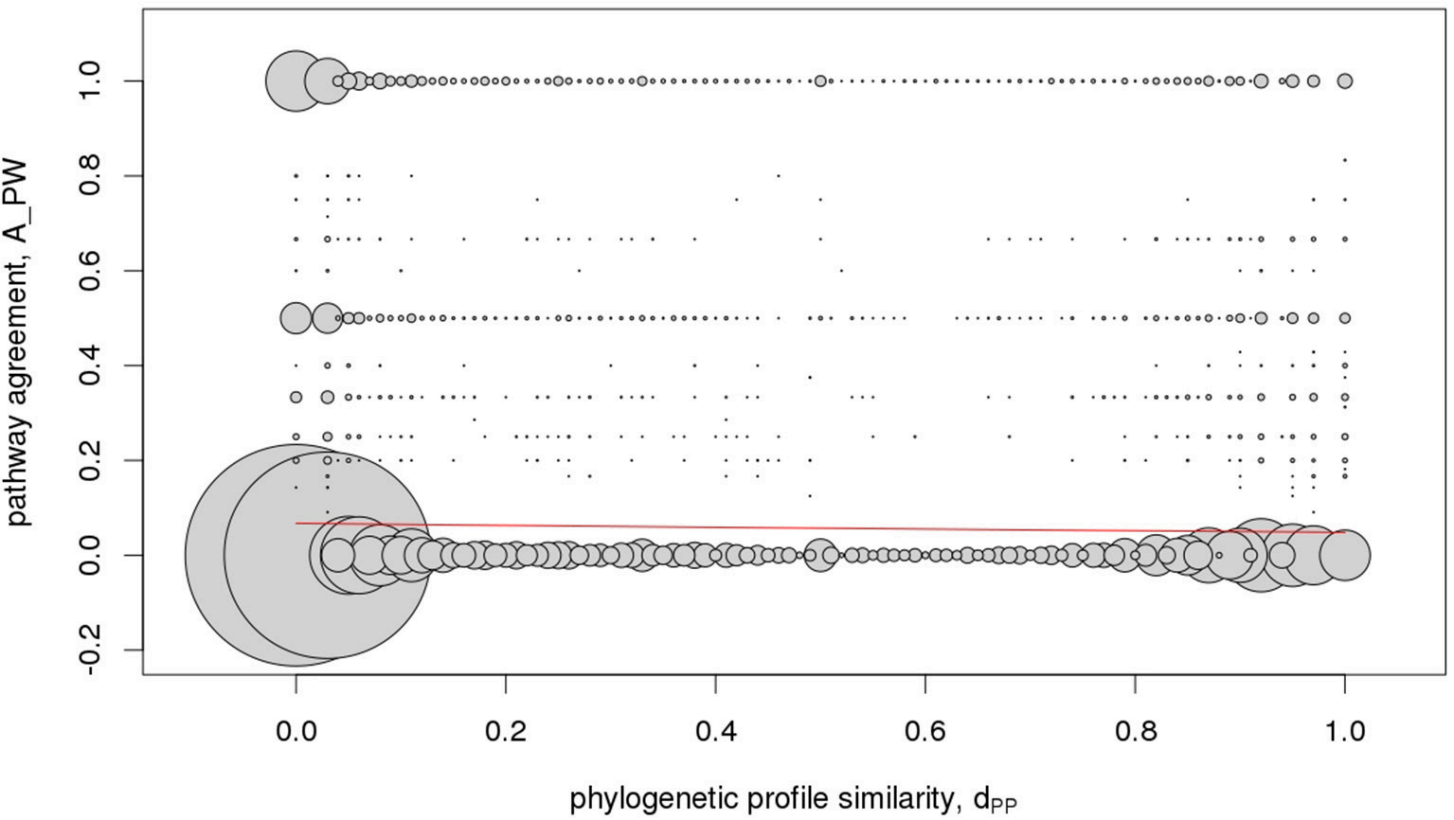
Metabolic pathway - phylogenetic profile correlation statistic. Pairwise agreement of pathway memberships, A_PW, of gene objects (families and singletons) in relation to their respective phylogenetic profile similarity, d_PP_. Because the underlying data are discrete resulting in many identical value-pairs, their respective frequency is illustrated by the area of the circles centered on the observed value-pairs. The red line signifies the logistic fit (see section Materials and Methods) and suggests that pathway similarity cannot be inferred on the basis of similar phylogenetic profiles of gene pairs.

To assess predictive value of phylogenetic profile similarity with regard to assigning two genes to either belong to the same or different pathway, we randomly drew two different gene objects from all 2,206 gene objects comprising 1,686 gene families and 520 singleton genes and determined their similarity with regard to phylogenetic profile and pathway assignments (see section Materials and Methods). If predictive, high phylogenetic profile similarity, reflected in our approach by values of d_PP_ (Equation 3) close to one, should indicate high agreement of pathway assignments of two gene objects, with A_PW-values (Equation 4) approaching one. However, as displayed in Figure 7, we observed no correlation of both similarity measures letting us conclude that given the available data and applied definitions, phylogenetic profile similarity is not predictive of pathway association.

**Figure 8:**
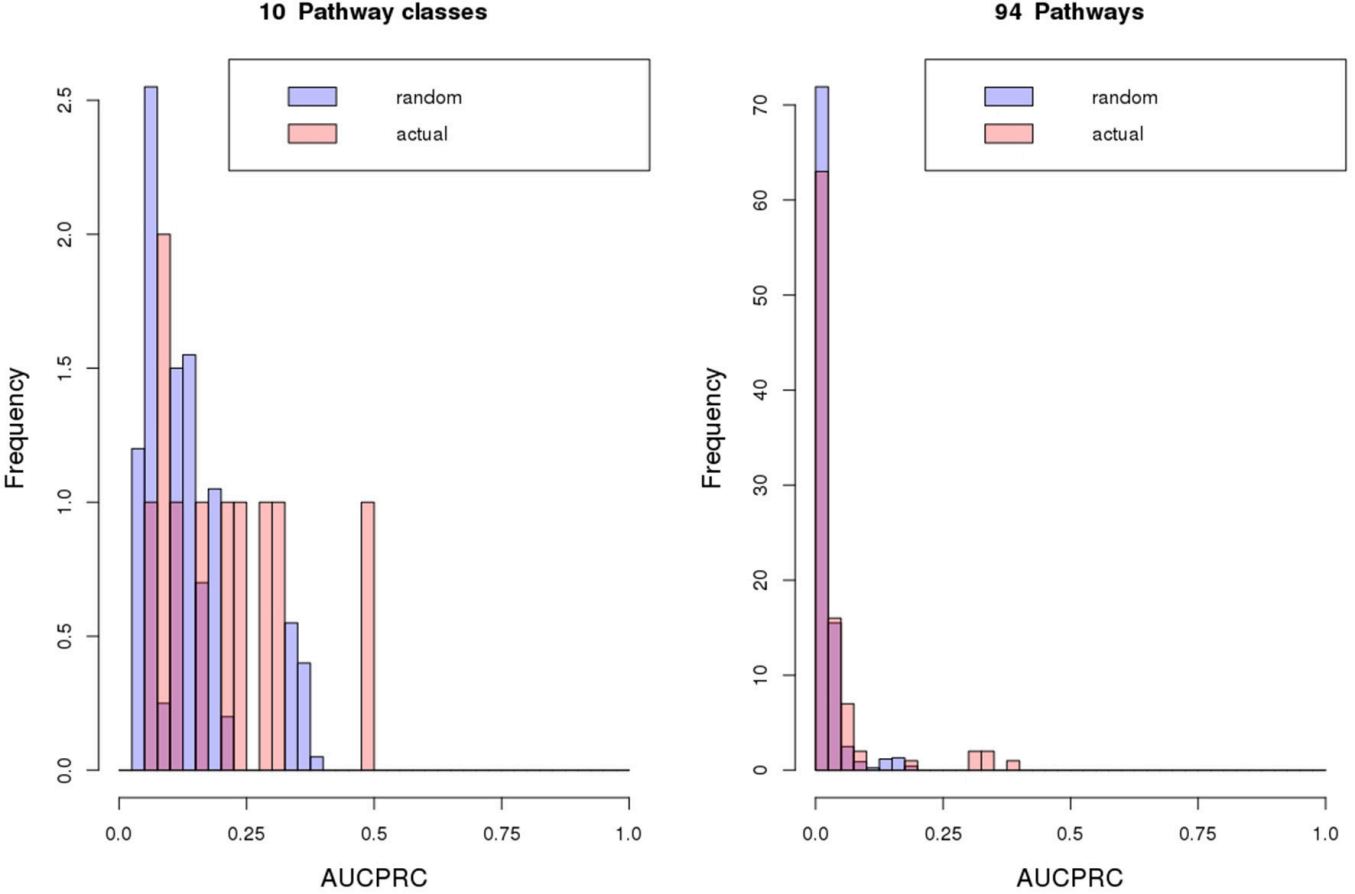
Classification results. Classification results of metabolic pathway class or map assignments of gene objects based on their phylogenetic profiles using Random Forest predictions as implemented in Clus-HMC. Performance is judged by the area under the precision-recall curve (AUCPRC). For the 100/20 randomized repeats performed for pathway map or class respectively, average AUCPRC distributions are plotted. Averaged over all random repeat runs, tests for statistical difference (Wilcoxon rank sum test) between actual and random AUCPRC value distributions yielded for pathway class: mean actual = 0.2, mean random = 0.13, *p* = 0.22, and for pathway map: mean actual = 0.04, mean random = 0.021, *p* = 0.0033. Clus-HMC was used allowing multiple and hierarchically organized labels per object with the hierarchy related to metabolism class and metabolism map.

Next, we treated the task of pathway assignment based on phylogenetic profiles as a machine learning problem. Using the Clus-HMC software package (see section Materials and Methods for details) that allows for hierarchical data structures as prediction targets (pathway class with detailed pathway maps in the next lower level) as well as allowing multiple labels (a gene can participate in more than one pathway), we aimed to predict metabolic class and pathway map for all gene families and singleton genes based on their phylogenetic profile. Approaching the prediction via a machine learning methodology (Random Forests) would possibly allow selected species to receive higher predictive value than treating all entries equally as done in the profile-profile comparison metric. In a cross-validation setting (out-of-bag error in Random Forest classification tree predictions), highest precision of prediction was achieved for central metabolism pathways when considering metabolism class (Table 4, Figure 8) and the detailed pathway maps “Photosynthesis,” “Pentose and glucuronate interconversions,” “Starch and sucrose metabolism,” “Pyrimidine metabolism,” and “Purine metabolism” (Table 5), again pathways associated with primary metabolism. Compared to randomly shuffled data, significantly better than random predictions were obtained at the pathway map level (*p* = 0.0033, Figure 8), whereas for class-level data, significance could not be established albeit the correct data corresponded to larger areas under the precision-recall curve (AUCPRC) than obtained for shuffled data (*p* = 0.22, Figure 8).

**Table 4:** Clus-HMC random forest prediction results of metabolic class based on phylogenetic profile of gene families and singleton genes.

**KEGG Metabolism class**	**NGF/S**	**AUCPRC**
Carbohydrate metabolism (CM)	549	0.478
Nucleotide metabolism (NM)	301	0.318
Energy metabolism (EM)	204	0.293
Amino acid metabolism (AAM)	292	0.246
Metabolism of cofactors and vitamins (MCV)	229	0.212
Lipid metabolism (LM)	207	0.151
**Biosynthesis of other secondary metabolites (BSM)**	106	0.110
Glycan biosynthesis and metabolism (GBM)	114	0.082
Metabolism of other amino acids (MOAA)	97	0.076
**Metabolism of terpenoids and polyketides (MTP)**	94	0.059

*Reported for the 10 metabolic classes are the number of gene families/ singletons (NGF/S) and the obtained areas under the prediction-recall curve (AUCPRC). The two classes corresponding to secondary metabolism processes are highlighted in bold-face font*.

**Table 5:** Clus-HMC random forest prediction results of metabolic pathway map based on phylogenetic profile of gene families and singleton genes.

**Pathway map number and name**	**NGF/S**	**Class**	**AUCPRC**
00195 Photosynthesis	30	EM	0.383
00040 Pentose and glucuronate interconversions	194	CM	0.340
00500 Starch and sucrose metabolism	292	CM	0.336
00240 Pyrimidine metabolism	325	NM	0.317
00230 Purine metabolism	367	NM	0.312

*Listed are the number of gene families/ singletons (NGF/S), the abbreviated metabolic class to which the map belongs, and the obtained areas under the prediction-recall curve (AUCPRC). Of all 94 maps, listed are those with AUCPRC > 0.25 as for those, predictions were significantly better than random (Figure 8). A complete list including all 94 pathways is provided as Supplementary Table 2*.

#### Phylogenetic profile similarity as an indicator of gene co-expression and protein-protein interactions

So far, we aimed to infer metabolic pathway relationships of genes via the similarity of their phylogenetic profiles. Next, we investigated whether phylogenetic profiles prove informative with regard to gene co-expression regulation of the encoded transcripts and physical interactions of their protein products. Like metabolic pathway membership, both types of associations can be taken as evidence of involvement in similar functional processes (Durek and Walther, 2008; Walther et al., 2010). As physical interactions (protein-protein interactions) represent direct associations, the rationale of phylogenetic profile similarity reflecting functional metabolic associations may become most apparent when testing them against protein-protein interactions. In these analyses, we focused on genes from the plant *A. thaliana* as rich experimental information on gene expression and protein-protein interactions are available. Specifically, we selected pairs of enzyme-encoding genes from Arabidopsis, retrieved their expression profiles from NASCArray, correlated them and also checked whether their protein products have been reported to interact (see section Materials and Methods for details). We then tested whether their association as judged by co-expression or physical interaction is correctly reflected by similarity of the phylogenetic profiles of the gene families to which the Arabidopsis genes belong. As this study focus on metabolic aspects, we considered enzyme encoding genes only.

**Figure 9:**
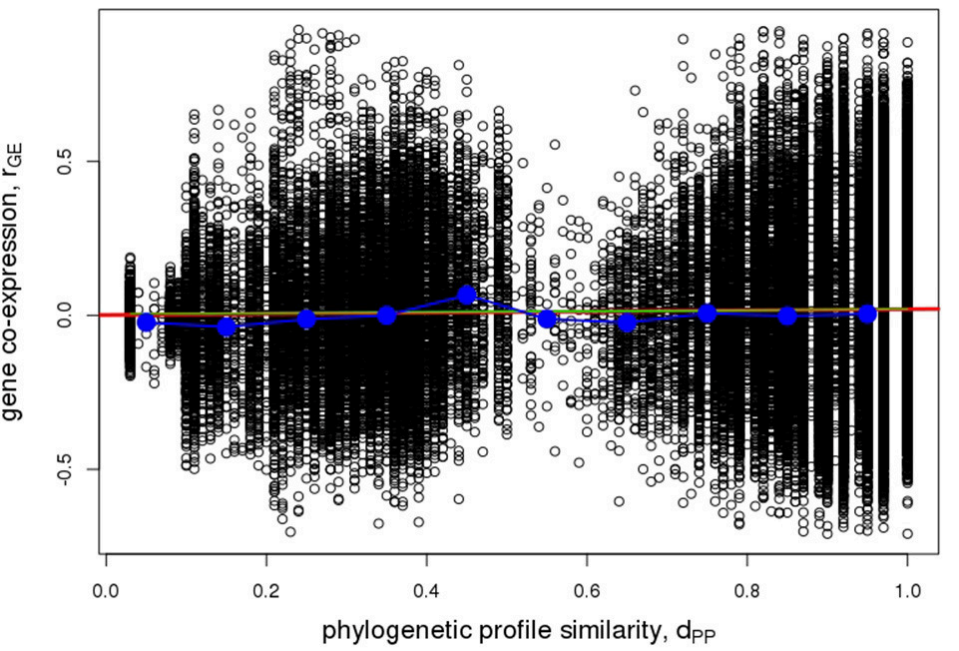
Pairwise gene co-expression statistic. Association of pairwise phylogenetic profile similarity, d_PP_, and co-expression of Arabidopsis gene pairs as judged by their pairwise Pearson correlation coefficient, r_GE_. Raw pairwise data are plotted as open circles, the red line indicates the linear regression line (*r* = 0.022, *p* = 2.9^*^10^−11^), the green line corresponds to a logistic fit virtually coinciding with the linear regression line, blue circles indicate median values of binned data (bin width = 0.1 d_PP_ units) connected by straight lines for visual guidance.

**Figure 10:**
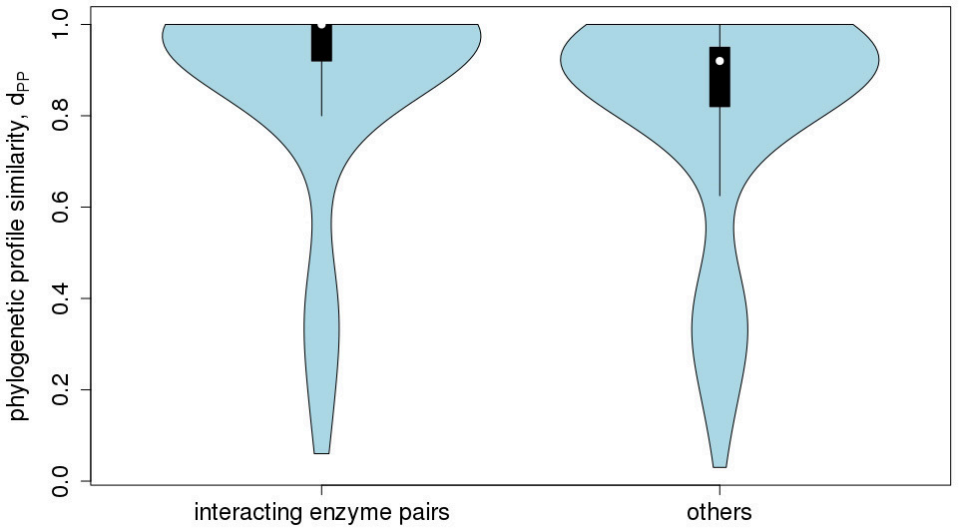
Protein-protein interaction statistic. Violin-plot of the frequency distribution of phylogenetic profile similarity values, d_PP_, of enzyme pairs encoded in *Arabidopsis thaliana* reported to physically interact (median = 1) to those pairs not reported to interact (median = 0.92, *p* < 2.2^*^10^−16^).

With regard to co-expression of Arabidopsis metabolic enzyme genes, no evidence of increasing profile-similarity being reflected by increased co-expression regulation was detected (*r* = 0.022, *p* = 2.9^*^10^−11^, Figure 9), albeit the correlation between the two distance measures proved significant, but due to the high number of value pairs. By contrast, a pronounced and statistically significant difference was found when testing for protein-protein interactions. Physically interacting enzymes were observed to be associated with genes whose phylogenetic profiles are more similar to one another (median value of d_PP_ = 1), than for non-interacting enzyme proteins (median value of d_PP_ = 0.92, *p* < 2.2^*^10^−16^, Figure 10). Thus, as argued above, direct interactions are indeed reflected by phylogenetic profile similarity, while gene co-expression, which includes gene pairs that operate in distant functional processes, is generally not.

## Discussion

In this study, we tested the applicability of phylogenetic profile similarity as an indicator of functional association between genes. Specifically, we aimed to determine whether patterns of correlated presence or absence of genes and their particular functions across different plant genomes can be used to infer metabolic pathway relationships. We focused on secondary metabolism as it is known that secondary metabolism exhibits pronounced confinements to particular species, a prerequisite for phylogenetic profiling to be informative and, thus, successful in predicting functional gene associations. We performed an array of analysis based on 39 plant genomes and gene functional annotation as available in KEGG. We approached answering the key questions of this study from various angles, first testing, whether genes assigned to known metabolic pathways show greater than expected agreement between their phylogenetic profiles. Then, we turned this approach around by asking whether pathway association can be predicted based on phylogenetic profiles. Lastly, we also tested whether phylogenetic profile similarity informs on gene co-expression and physical interactions of their encoded protein product.

With regard to the central question to assign metabolic pathway relationships, the results of our feasibility study were largely negative. Phylogenetic profile similarity did not prove informative with regard to metabolic pathway relationships. More precisely, no specific predictions could be made. Correct pathway associations were predicted for primary metabolism pathways only (Figure 8, Tables 2–5). Thus, the methodology correctly predicted essential processes based on presence/absence calls of genes across genomes, which however, involve several pathways. Thus, specificity is lost. Assigning secondary pathway relationships proved not possible, nor was gene co-expression correlated with phylogenetic profile similarity (Figure 9). The only, but very notable, exception were physical interactions between protein products (Figure 10). Here, phylogenetic profiles proved of predictive value. Direct physical interactions of enzyme proteins are reflected by an increased phylogenetic profile similarity of genes encoding them. Thus, rather than extending to associations of genes at larger functional distances captured by the assignment of genes to a common pathway, phylogenetic profiles proved predictive only for short range, in fact, direct functional interactions involving physical contacts.

The largely negative results of this feasibility study call for a critical review of the study design, used data, and applied methodology. Critical aspects concern the selection of plant species, the assignment of genes to gene families as the critical step to establishing presence or absence of particular gene functions in genomes, and the richness and accuracy of metabolic pathway annotation, specifically concerning secondary metabolism pathways, as well as the notion of distinct metabolic pathways as a suitable abstraction of functional interactions between enzymes.

### Selection of plant species

In essence, the phylogenetic profiling approach relies on correlations of vectors (binary presence/absence calls of genes or their respectively encoded functions). Thus, as with any correlation measure, confidence of a significant correlation should increase with length of the vectors; i.e., the number of value pairs, or genomes in our case, to be compared. Therefore, it seems desirable to include as many genomes/plant species as possible. When testing for the required minimal number of genomes to be included in phylogenetic profiling studies, prediction results were plateauing beyond 100 included genomes with an additional importance associated to increased phylogenetic diversity rather than bare counts of genomes (Škunca and Dessimoz, 2015). Similarly, it was reported that aside from including many genomes, their selection (e.g., with regard to kingdom) matters as well and that, furthermore, the selection of genomes may have different bearings on the predictability of different pathways (Jothi et al., 2007).

Here, we included 39 plant species covering a broad spectrum of plant species from algae to higher plants (Table 1). Despite being a comparably promising number of genomes as judged by the reported 100 species optimum, evidently, further increasing this number of species would be desirable, but is dependent on the availability of sequenced plant genomes, which is likely to rapidly grow given the progress in sequencing technologies. Alternatively, we could have chosen to expand the number of considered genomes by including non-plant species. However, as we were specifically interested in plant secondary metabolism, which is (largely) absent in non-plant species, we opted against it. Including non-plant species would likely have yielded strong predictions for assigning relationships of genes present in plants only. Yet, we believe, discerning plant-only genes and functions can be achieved based on single gene comparison alone without imposing correlated inheritance patterns needed specifically to discern functional relationships. By including non-plant genomes would have yielded unspecific results that the interaction is confined to the plant kingdom.

### Assignment of genes to gene families as the critical step to establishing presence or absence of particular gene functions in genomes

Most critically, phylogenetic profiling depends on the correct assignment of a gene being present or not. More precisely, it needs to be decided whether the particular function observed to be performed in a reference species can be performed in another species, and therefore, a homologous gene would be found encoded in it.

We operated under the assumption that sequence-similar enzymes perform similar or identical functions. Hence, the presence or absence of a particular enzymatic activity in a given genome can be determined based on sequence similarity to an annotated reference gene. While it has been shown that sequence similarity is indeed a good predictor of similar protein structure and, thus, function (Sander and Schneider, 1991), and that above 40% sequence identity functional differences are unlikely (Lo Conte et al., 2002; Orengo et al., 2002), contradicting examples have also been described. For example, proteins with high sequence similarity to photosynthesis related genes were found in non-photosynthetically active organisms invalidating any sequence-based functional assignment to photosynthetic processes (Ashkenazi et al., 2012). More generally, early conclusions suggesting relatively low sequence identity thresholds as sufficient for a reliable functional annotation transfer were called into question by pointing to possible database biases (Rost, 2002). Following up on this study, Tian and Skolnick showed that at 40% sequence identity, transfer of enzymatic function at the level of the first three EC number digits is reliable. However, to predict all four digits, 60% sequence identity levels are necessary to achieve 90% accuracy (Tian and Skolnick, 2003). Therefore, it appears surprising that we obtained best agreement of gene family clustering and enzymatic function annotation at 30%, and not 70%, sequence identity. We believe, this apparent contradiction is explained by realizing that Tian and Skolnick (2003) excluded all computational predictions, while we included them. Therefore, our results will depend on the sequence comparison thresholds applied by the original genome curators, which very likely included more generous sequence identity threshold levels. And as our plant dataset contains many species that have been less intensively studied experimentally, computational annotations will form the basis of many functional assignments. Furthermore, it is clear that sequence identity across the entire sequence can only be on average a good predictor of function as even single amino acid mutation may suffice to alter an enzyme's function, for example with regard to substrate specificity (Khersonsky et al., 2006).

For generating gene families, we also tested OrthoFinder (Emms and Kelly, 2015) as well as applied community detection algorithms to the networks based on sequence-comparison based networks to identify sub-clusters of genes, which could be regarded individual gene families. However, those attempts did not yield qualitatively different results than reported here based on pairwise sequence-identity thresholds assignments.

### Richness and accuracy of metabolic pathway annotation

We specifically aimed at exploiting phylogenetic profiling for the identification of genes commonly involved in specific plant secondary metabolism pathways. As secondary metabolism pathways are known to occur specifically in particular species (Hartmann, 1996; Higashi and Saito, 2013), the requirements of phylogenetic profiling seem ideally met. However, for secondary metabolism pathways, low statistical concordance of member gene profiles (Tables 2, 3, Supplementary Table 1) and poor prediction results (Tables 4, 5, Supplementary Table 2) were obtained.

An obvious and serious limitation of our study lies in the paucity of experimentally annotated, and most importantly, species-specific annotation of secondary metabolism pathways and their associated genes explained by the experimentally challenges to deduce pathways and involved genes. To large degree, enzymatic pathway annotation relies on homology-based transfer of annotations from model species with *A. thaliana* being the most significant one. While *A. thaliana* was found to exhibit a richer than expected secondary metabolism (D'Auria and Gershenzon, 2005), relying on a single or few well characterized species will naturally limit the ability to test the predictive value phylogenetic profiling. It is important to note that the limitations concern the testability of predictions. Predictions may still be correct, but it is not possible to compare them to known annotations. Thus, to further develop and exploit concepts phylogenetic profiling, an enlarged set of functionally characterized and specific plant pathway genes in diverse plant species would be highly desirable.

In our analyses, we used annotated enzyme genes only. In applying phylogenetic profiling to novel genomes, of course, it would not be known *a priori* whether a gene codes for enzyme. However, this study was designed specifically as a feasibility study such that a comparison to true (within the limits of its accuracy) functional assignments can be made. In praxis, classical sequence-comparison based methods could be used to establish enzymatic functionalities first, which however, would also allow assigning novel genes to pathways if a corresponding annotation is available. It was exactly the aim of this study to test, whether such functional assignments can be made based on phylogenetic profile similarity alone; i.e., without requiring detailed annotation knowledge. Unfortunately, this promise did not materialize.

We based our metabolic pathway annotation information on KEGG. While KEGG is highly regarded, alternative databases focusing on plant species have been developed (Grafahrend-Belau et al., 2012) amongst which the Plant Metabolic Network (PMN, aka PlantCyc, www.plantcyc.org) represents another large-scale plant-metabolism-centric data resource. For comparison, we also performed the phylogenetic profile enrichment analysis using PlantCyc data (Supplementary Table 3). While a larger set of 241 pathways was available for analysis, only five proved to be significant with regard to enriched phylogenetic profile similarity. In close agreement with the KEGG results, the “Calvin-Benson-Bassham cycle” pathway (Benjamini-Hochberg corrected p_BH_ < 0.001) turned out to be most significant (using KEGG data, it was ranked fifth and designated as “Carbon fixation in photosynthetic organisms,” followed by “brassinosteroids inactivation” (p_BH_ < 0.001), “oryzalide A biosynthesis” (p_BH_ = 0.048), “5-aminoimidazole ribonucleotide biosynthesis II” (p_BH_ = 0.048, and “L-arginine biosynthesis II (acetyl cycle),” p_BH_ = 0.048). Thus, the more detailed pathway description available in PlantCyc did not result in increased associations, even though it needs to be considered that the larger set sizes (241 vs. 94 in KEGG) causes a more pronounced multiple testing correction effect. Nonetheless, we conclude that the results reported here are not specific to KEGG, but point to a general weakness of the approach and the current data availability.

### Notion of distinct metabolic pathways as a suitable abstraction of functional interactions between enzymes

Primarily, we based functional associations of genes objects on their occurrence in the same KEGG pathway class or detailed pathway map. Thus, we treated pathways as isolated containers with all genes in them exhibiting a functional relationship irrespective of the actual number of reactions steps between them. Using this definition, largely poor statistical prediction results were obtained. By contrast, when inspecting direct and physical interaction between enzymes, phylogenetic profiles proved highly informative (Figure 10). As, often, physical interactions indicate immediate metabolic reaction relationships (Durek and Walther, 2008), this result can be taken as positive study result pointing also to the importance of metabolic pathway distance between enzymes. Therefore, phylogenetic profile similarity may be taken as a suitable filtering to identify true protein-protein interactions in experimental or prediction interaction sets. Apparently, with larger metabolic pathway distance, predictive value of phylogenetic profiles decays quickly. As a conclusion, switching from pathway containers to a network-based distance between enzyme genes seems in order. This would also address another obvious limitation of pathway containers. They treat metabolic relationship as isolated sub-pathways, such that genes are either involved in the same process or not at all associated. This is illustrated also in Figure 3 for the diterpenoid biosynthesis pathway. While some genes in this pathway container (map) or found in many species, branch-reactions have a narrow species-occurrence pattern. Treating all genes identically in this map seems incorrect and will lead to wrong conclusions. Evidently, a distance metric that captures the true metabolic pathway distance, for example, shortest paths (Durek and Walther, 2008; Walther et al., 2010), would be preferable. However, despite these severe limitations, we still regard the approach presented in this study a valid first step toward attaining the goal of correct functional association prediction of enzyme genes.

## Conclusions

In conclusion, phylogenetic profile similarities proved insensitive to yield reliable predictions of associations of genes at the level of metabolic pathway classes and maps, but were informative with regard to physical interactions of encoded enzyme proteins. This study underlines the need to expand our experimental knowledge of secondary metabolism pathway across different plant species before a final judgment of the applicability of phylogenetic profiles can be made. It also critically reflects on the concept of assigning genes as functionally linked via pathway memberships alone. Instead, a network-based distance metric appears desirable. The positive correlation of profiles with physical interactions opens the possibility to use phylogenetic profiling as a filtering step to identify true protein-protein interactions from candidate interaction sets.

## Author contributions

DW conceived the study, SW and DW designed the study, interpreted the results, and wrote the manuscript. All computations were performed by SW, except for the Clus-HMC predictions and gene co-expression analyses performed by DW.

### Conflict of interest statement

The authors declare that the research was conducted in the absence of any commercial or financial relationships that could be construed as a potential conflict of interest.
