# Supplementary material for: Metabolic Pathway Assignment of Plant Genes based on Phylogenetic Profiling–A Feasibility Study
Source: Front Plant Sci. 2017 Oct 27;8:1831. doi: 10.3389/fpls.2017.01831 (PMC5664361; doi:10.3389/fpls.2017.01831)
Supplement: Supplementary file 1 [file Table1.DOCX]

Supplementary Table 1. Statistics of metabolism pathway assignments and phylogenetic profile identities of gene families and singletons. For all KEGG pathways considered in this study and given by their KEGG map number and name, table lists the metabolism class to which pathway belongs indicated by their abbreviations introduced in Table 1, the number of gene families or singleton genes (referred to as gene objects, N_GF/S_) annotated to them, the fraction of all profile-profile comparisons among all N_GF/S_ gene objects yielding identical profiles within a pathway (F_pw_, Eq.1), the fraction of all profile-profile comparisons of N_GF/S_ gene objects yielding identical profiles within and to gene objects outside a pathway (F_all_, Eq.2), the resulting fold enrichment (E= F_pw_/F_all_) of identical phylogenetic profiles within a pathway relative to expectation, and associated Benjamini-Hochberg corrected empirical p-value based on 10,000 random assignments. Results are based on Network30-based gene family assignments (see Methods). Pathways belonging to secondary metabolism classes Biosynthesis of other secondary metabolites (BSM) and Metabolism of terpenoids and polyketides (MTP) are highlighted using bold face font. Pathways are sorted in ascending order of p-value.

| Pathway map number and name | Class | N_GF/S_ | F_pw_ | F_all_ | E = F_pw_/F_all_ | Adjusted p-value |
| --- | --- | --- | --- | --- | --- | --- |
| 00450 Selenocompound metabolism | MOAA | 15 | 0.152 | 0.037 | 4.165 | <0.001 |
| 00020 Citrate cycle (TCA cycle) | CM | 31 | 0.146 | 0.035 | 4.145 | <0.001 |
| 00920 Sulfur metabolism | EM | 23 | 0.142 | 0.035 | 4.014 | <0.001 |
| 00660 C5-Branched dibasic acid metabolism | CM | 15 | 0.152 | 0.039 | 3.958 | <0.001 |
| 00280 Valine. leucine and isoleucine degradation | AAM | 26 | 0.120 | 0.036 | 3.377 | <0.001 |
| 00710 Carbon fixation in photosynthetic organisms | EM | 70 | 0.065 | 0.026 | 2.498 | <0.001 |
| 00250 Alanine, aspartate and glutamate metabolism | AAM | 43 | 0.063 | 0.026 | 2.467 | <0.001 |
| 00400 Phenylalanine, tyrosine and tryptophan biosynthesis | AAM | 49 | 0.061 | 0.028 | 2.188 | <0.001 |
| 00010 Glycolysis / Gluconeogenesis | CM | 105 | 0.050 | 0.024 | 2.134 | <0.001 |
| 00260 Glycine, serine and threonine metabolism | AAM | 75 | 0.049 | 0.024 | 2.068 | <0.001 |
| 00620 Pyruvate metabolism | CM | 72 | 0.047 | 0.024 | 2.000 | <0.001 |
| 00240 Pyrimidine metabolism | NM | 325 | 0.023 | 0.014 | 1.670 | <0.001 |
| 00300 Lysine biosynthesis | AAM | 16 | 0.125 | 0.034 | 3.682 | 0.006 |
| 00650 Butanoate metabolism | CM | 25 | 0.097 | 0.035 | 2.746 | 0.006 |
| 00640 Propanoate metabolism | CM | 41 | 0.070 | 0.029 | 2.428 | 0.006 |
| 00270 Cysteine and methionine metabolism | AAM | 70 | 0.053 | 0.025 | 2.180 | 0.006 |
| 00480 Glutathione metabolism | MOAA | 42 | 0.050 | 0.023 | 2.152 | 0.010 |
| 00030 Pentose phosphate pathway | CM | 63 | 0.040 | 0.024 | 1.684 | 0.010 |
| 00071 Fatty acid degradation | LM | 15 | 0.114 | 0.034 | 3.365 | 0.015 |
| 00945 Stilbenoid, diarylheptanoid and gingerol biosynthesis | **BSM** | 12 | 0.152 | 0.012 | 12.553 | 0.024 |
| 00220 Arginine biosynthesis | AAM | 41 | 0.041 | 0.022 | 1.865 | 0.030 |
| 00290 Valine, leucine and isoleucine biosynthesis | AAM | 35 | 0.047 | 0.026 | 1.820 | 0.030 |
| 00630 Glyoxylate and dicarboxylate metabolism | CM | 74 | 0.035 | 0.022 | 1.613 | 0.033 |
| 00051 Fructose and mannose metabolism | CM | 65 | 0.034 | 0.021 | 1.616 | 0.035 |
| 00860 Porphyrin and chlorophyll metabolism | MCV | 63 | 0.034 | 0.021 | 1.638 | 0.041 |
| 00410 beta-Alanine metabolism | MOAA | 24 | 0.047 | 0.027 | 1.740 | 0.047 |
| 00230 Purine metabolism | NM | 367 | 0.021 | 0.014 | 1.487 | 0.049 |
| 00310 Lysine degradation | AAM | 13 | 0.077 | 0.037 | 2.060 | 0.050 |
| 00760 Nicotinate and nicotinamide metabolism | MCV | 22 | 0.048 | 0.022 | 2.214 | 0.052 |
| 00904 Diterpenoid biosynthesis | **MTP** | 7 | 0.143 | 0.012 | 11.626 | 0.069 |
| 00380 Tryptophan metabolism | AAM | 35 | 0.039 | 0.022 | 1.730 | 0.091 |
| 00941 Flavonoid biosynthesis | **BSM** | 14 | 0.066 | 0.014 | 4.695 | 0.097 |
| 00790 Folate biosynthesis | MCV | 30 | 0.039 | 0.020 | 2.003 | 0.097 |
| 00330 Arginine and proline metabolism | AAM | 36 | 0.032 | 0.022 | 1.464 | 0.113 |
| 00903 Limonene and pinene degradation | **MTP** | 10 | 0.067 | 0.009 | 7.296 | 0.142 |
| 00340 Histidine metabolism | AAM | 18 | 0.039 | 0.019 | 2.016 | 0.201 |
| 00040 Pentose and glucuronate interconversions | CM | 194 | 0.020 | 0.010 | 1.985 | 0.203 |
| 00562 Inositol phosphate metabolism | CM | 53 | 0.025 | 0.020 | 1.232 | 0.203 |
| 00073 Cutin, suberine and wax biosynthesis | LM | 26 | 0.031 | 0.015 | 2.052 | 0.221 |
| 00592 alpha-Linolenic acid metabolism | LM | 27 | 0.031 | 0.019 | 1.620 | 0.221 |
| 01040 Biosynthesis of unsaturated fatty acids | LM | 12 | 0.045 | 0.024 | 1.930 | 0.241 |
| 00670 One carbon pool by folate | MCV | 45 | 0.024 | 0.019 | 1.256 | 0.244 |
| 00130 Ubiquinone and other terpenoid-quinone biosynthesis | MCV | 23 | 0.028 | 0.018 | 1.549 | 0.289 |
| 00100 Steroid biosynthesis | LM | 16 | 0.033 | 0.014 | 2.361 | 0.325 |
| 00430 Taurine and hypotaurine metabolism | MOAA | 11 | 0.036 | 0.023 | 1.601 | 0.336 |
| 00770 Pantothenate and CoA biosynthesis | MCV | 45 | 0.021 | 0.018 | 1.180 | 0.378 |
| 00750 Vitamin B6 metabolism | MCV | 18 | 0.026 | 0.011 | 2.430 | 0.410 |
| 00520 Amino sugar and nucleotide sugar metabolism | CM | 119 | 0.018 | 0.015 | 1.234 | 0.423 |
| 00730 Thiamine metabolism | MCV | 18 | 0.026 | 0.022 | 1.180 | 0.430 |
| 00900 Terpenoid backbone biosynthesis | **MTP** | 33 | 0.021 | 0.013 | 1.586 | 0.437 |
| 00910 Nitrogen metabolism | EM | 30 | 0.021 | 0.018 | 1.133 | 0.437 |
| 00053 Ascorbate and aldarate metabolism | CM | 24 | 0.022 | 0.019 | 1.143 | 0.463 |
| 00740 Riboflavin metabolism | MCV | 17 | 0.022 | 0.023 | 0.952 | 0.550 |
| 00565 Etherlipid metabolism | LM | 21 | 0.019 | 0.009 | 2.132 | 0.583 |
| 00460 Cyanoamino acid metabolism | MOAA | 28 | 0.019 | 0.018 | 1.017 | 0.591 |
| 00360 Phenylalanine metabolism | AAM | 24 | 0.018 | 0.019 | 0.942 | 0.591 |
| 00511 Other glycan degradation | GBM | 40 | 0.015 | 0.011 | 1.364 | 0.668 |
| 00052 Galactose metabolism | CM | 92 | 0.015 | 0.013 | 1.164 | 0.692 |
| 00062 Fatty acid elongation | LM | 39 | 0.015 | 0.012 | 1.201 | 0.724 |
| 00350 Tyrosine metabolism | AAM | 28 | 0.016 | 0.014 | 1.123 | 0.724 |
| 00780 Biotin metabolism | MCV | 11 | 0.018 | 0.016 | 1.113 | 0.813 |
| 00061 Fatty acid biosynthesis | LM | 32 | 0.014 | 0.018 | 0.784 | 0.813 |
| 00500 Starch and sucrose metabolism | CM | 292 | 0.015 | 0.011 | 1.368 | 0.819 |
| 00603 Glycosphingolipid biosynthesis - globo series | GBM | 12 | 0.015 | 0.007 | 2.133 | 0.881 |
| 00604 Glycosphingolipid biosynthesis - ganglio series | GBM | 32 | 0.012 | 0.011 | 1.132 | 0.885 |
| 00600 Sphingolipid metabolism | LM | 58 | 0.013 | 0.014 | 0.930 | 0.892 |
| 00906 Carotenoid biosynthesis | **MTP** | 20 | 0.011 | 0.012 | 0.842 | 0.946 |
| 00514 Other types of O-glycan biosynthesis | GBM | 34 | 0.011 | 0.010 | 1.090 | 0.948 |
| 00531 Glycosaminoglycan degradation | GBM | 34 | 0.011 | 0.011 | 0.981 | 0.948 |
| 00561 Glycerolipid metabolism | LM | 50 | 0.011 | 0.012 | 0.931 | 0.948 |
| 00195 Photosynthesis | EM | 30 | 0.005 | 0.002 | 2.238 | 1.000 |
| 00190 Oxidative phosphorylation | EM | 112 | 0.011 | 0.009 | 1.199 | 1.000 |
| 00940 Phenylpropanoid biosynthesis | **BSM** | 65 | 0.008 | 0.008 | 0.992 | 1.000 |
| 00510 N-Glycan biosynthesis | GBM | 36 | 0.010 | 0.010 | 0.953 | 1.000 |
| 00564 Glycerophospholipid metabolism | LM | 64 | 0.009 | 0.011 | 0.837 | 1.000 |
| 00563 Glycosylphosphatidylinositol(GPI)-anchor biosynthesis | GBM | 25 | 0.003 | 0.004 | 0.822 | 1.000 |
| 00072 Synthesis and degradation of ketone bodies | LM | 8 | 0.000 | 0.010 | 0.000 | 1.000 |
| 00196 Photosynthesis - antenna proteins | EM | 3 | 0.000 | 0.000 | 0.000 | 1.000 |
| 00471 D-Glutamine and D-glutamate metabolism | MOAA | 9 | 0.000 | 0.006 | 0.000 | 1.000 |
| 00590 Arachidonic acid metabolism | LM | 9 | 0.000 | 0.004 | 0.000 | 1.000 |
| 00591 Linoleic acid metabolism | LM | 6 | 0.000 | 0.002 | 0.000 | 1.000 |
| 00785 Lipoic acid metabolism | MCV | 10 | 0.000 | 0.012 | 0.000 | 1.000 |
| 00901 Indole alkaloid biosynthesis | **BSM** | 2 | 0.000 | 0.019 | 0.000 | 1.000 |
| 00902 Monoterpenoid biosynthesis | **MTP** | 2 | 0.000 | 0.006 | 0.000 | 1.000 |
| 00905 Brassinosteroid biosynthesis | **MTP** | 2 | 0.000 | 0.016 | 0.000 | 1.000 |
| 00908 Zeatin biosynthesis | **MTP** | 11 | 0.000 | 0.012 | 0.000 | 1.000 |
| 00909 Sesquiterpenoid and triterpenoid biosynthesis | **MTP** | 3 | 0.000 | 0.003 | 0.000 | 1.000 |
| 00942 Anthocyanin biosynthesis | **BSM** | 2 | 0.000 | 0.004 | 0.000 | 1.000 |
| 00944 Flavone and flavonol biosynthesis | **BSM** | 3 | 0.000 | 0.010 | 0.000 | 1.000 |
| 00966 Glucosinolate biosynthesis | **BSM** | 3 | 0.000 | 0.002 | 0.000 | 1.000 |
| 00232 Caffeine metabolism | **BSM** | 1 | NA | 0.030 | NA | 1.000 |
| 00523 Polyketide sugar unit biosynthesis | **MTP** | 1 | NA | 0.079 | NA | 1.000 |
| 00960 Tropane, piperidine and pyridine alkaloid biosynthesis | **BSM** | 1 | NA | 0.079 | NA | 1.000 |
| 00965 Betalain biosynthesis | **BSM** | 1 | NA | 0.000 | NA | 1.000 |
