# Supplementary material for: Metabolic Pathway Assignment of Plant Genes based on Phylogenetic Profiling–A Feasibility Study
Source: Front Plant Sci. 2017 Oct 27;8:1831. doi: 10.3389/fpls.2017.01831 (PMC5664361; doi:10.3389/fpls.2017.01831)
Supplement: Supplementary file 2 [file Table2.DOCX]

**Supplementary Table 2. Clus-HMC Prediction results for all 94 metabolism pathways considered in this study.**

| Pathway map number and name | NGF/S | Class | AUCPRC |
| --- | --- | --- | --- |
| 00195 Photosynthesis | 30 | EM | 0.383 |
| 00040 Pentose and glucuronate interconversions | 194 | CM | 0.340 |
| 00500 Starch and sucrose metabolism | 292 | CM | 0.336 |
| 00240 Pyrimidine metabolism | 325 | NM | 0.317 |
| 00230 Purine metabolism | 367 | NM | 0.312 |
| 00190 Oxidative phosphorylation | 112 | EM | 0.175 |
| 00860 Porphyrin and chlorophyll metabolism | 63 | MCV | 0.093 |
| 00520 Amino sugar and nucleotide sugar metabolism | 119 | CM | 0.084 |
| 00250 Alanine. aspartate and glutamate metabolism | 43 | AAM | 0.072 |
| 00010 Glycolysis / Gluconeogenesis | 105 | CM | 0.065 |
| 00710 Carbon fixation in photosynthetic organisms | 70 | EM | 0.065 |
| 00471 D-Glutamine and D-glutamate metabolism | 9 | MOAA | 0.064 |
| 00940 Phenylpropanoid biosynthesis | 65 | BSM | 0.061 |
| 00052 Galactose metabolism | 92 | CM | 0.059 |
| 00220 Arginine biosynthesis | 41 | AAM | 0.055 |
| 00945 Stilbenoid, diarylheptanoid and gingerol biosynthesis | 12 | BSM | 0.048 |
| 00100 Steroid biosynthesis | 16 | LM | 0.046 |
| 00630 Glyoxylate and dicarboxylate metabolism | 74 | CM | 0.043 |
| 00620 Pyruvate metabolism | 72 | CM | 0.042 |
| 00260 Glycine. serine and threonine metabolism | 75 | AAM | 0.042 |
| 00270 Cysteine and methionine metabolism | 70 | AAM | 0.040 |
| 00564 Glycerophospholipid metabolism | 64 | LM | 0.038 |
| 00640 Propanoate metabolism | 41 | CM | 0.036 |
| 00051 Fructose and mannose metabolism | 65 | CM | 0.035 |
| 00510 N-Glycan biosynthesis | 36 | GBM | 0.034 |
| 00400 Phenylalanine. tyrosine and tryptophan biosynthesis | 49 | AAM | 0.032 |
| 00020 Citrate cycle (TCA cycle) | 31 | CM | 0.030 |
| 00062 Fatty acid elongation | 39 | LM | 0.028 |
| 00760 Nicotinate and nicotinamide metabolism | 22 | MCV | 0.027 |
| 00600 Sphingolipid metabolism | 58 | LM | 0.025 |
| 00030 Pentose phosphate pathway | 63 | CM | 0.025 |
| 00430 Taurine and hypotaurine metabolism | 11 | MOAA | 0.025 |
| 00900 Terpenoid backbone biosynthesis | 33 | MTP | 0.024 |
| 00903 Limonene and pinene degradation | 10 | MTP | 0.024 |
| 00770 Pantothenate and CoA biosynthesis | 45 | MCV | 0.023 |
| 00561 Glycerolipid metabolism | 50 | LM | 0.023 |
| 00514 Other types of O-glycan biosynthesis | 34 | GBM | 0.023 |
| 00562 Inositol phosphate metabolism | 53 | CM | 0.023 |
| 00330 Arginine and proline metabolism | 36 | AAM | 0.022 |
| 00511 Other glycan degradation | 40 | GBM | 0.022 |
| 00563 Glycosylphosphatidylinositol(GPI)-anchor biosynthesis | 25 | GBM | 0.021 |
| 00480 Glutathione metabolism | 42 | MOAA | 0.020 |
| 00592 alpha-Linolenic acid metabolism | 27 | LM | 0.020 |
| 00670 One carbon pool by folate | 45 | MCV | 0.020 |
| 00380 Tryptophan metabolism | 35 | AAM | 0.020 |
| 00966 Glucosinolate biosynthesis | 3 | BSM | 0.020 |
| 00790 Folate biosynthesis | 30 | MCV | 0.019 |
| 00590 Arachidonic acid metabolism | 9 | LM | 0.018 |
| 00280 Valine. leucine and isoleucine degradation | 26 | AAM | 0.017 |
| 00910 Nitrogen metabolism | 30 | EM | 0.017 |
| 00290 Valine. leucine and isoleucine biosynthesis | 35 | AAM | 0.017 |
| 00531 Glycosaminoglycan degradation | 34 | GBM | 0.017 |
| 00073 Cutin. suberine and wax biosynthesis | 26 | LM | 0.017 |
| 00650 Butanoate metabolism | 25 | CM | 0.016 |
| 00071 Fatty acid degradation | 15 | LM | 0.016 |
| 00450 Selenocompound metabolism | 15 | MOAA | 0.016 |
| 00660 C5-Branched dibasic acid metabolism | 15 | CM | 0.016 |
| 00061 Fatty acid biosynthesis | 32 | LM | 0.016 |
| 00604 Glycosphingolipid biosynthesis - ganglio series | 32 | GBM | 0.016 |
| 00460 Cyanoamino acid metabolism | 28 | MOAA | 0.016 |
| 00130 Ubiquinone and other terpenoid-quinone biosynthesis | 23 | MCV | 0.015 |
| 00350 Tyrosine metabolism | 28 | AAM | 0.015 |
| 00906 Carotenoid biosynthesis | 20 | MTP | 0.015 |
| 00740 Riboflavin metabolism | 17 | MCV | 0.015 |
| 00920 Sulfur metabolism | 23 | EM | 0.014 |
| 00565 Etherlipid metabolism | 21 | LM | 0.014 |
| 01040 Biosynthesis of unsaturated fatty acids | 12 | LM | 0.013 |
| 00340 Histidine metabolism | 18 | AAM | 0.013 |
| 00750 Vitamin B6 metabolism | 18 | MCV | 0.013 |
| 00730 Thiamine metabolism | 18 | MCV | 0.013 |
| 00941 Flavonoid biosynthesis | 14 | BSM | 0.013 |
| 00785 Lipoic acid metabolism | 10 | MCV | 0.013 |
| 00053 Ascorbate and aldarate metabolism | 24 | CM | 0.013 |
| 00300 Lysine biosynthesis | 16 | AAM | 0.012 |
| 00410 beta-Alanine metabolism | 24 | MOAA | 0.012 |
| 00360 Phenylalanine metabolism | 24 | AAM | 0.010 |
| 00904 Diterpenoid biosynthesis | 7 | MTP | 0.009 |
| 00603 Glycosphingolipid biosynthesis - globo series | 12 | GBM | 0.008 |
| 00944 Flavone and flavonol biosynthesis | 3 | BSM | 0.007 |
| 00908 Zeatin biosynthesis | 11 | MTP | 0.007 |
| 00310 Lysine degradation | 13 | AAM | 0.006 |
| 00960 Tropane. piperidine and pyridine alkaloid biosynthesis | 1 | BSM | 0.005 |
| 00780 Biotin metabolism | 11 | MCV | 0.004 |
| 00942 Anthocyanin biosynthesis | 2 | BSM | 0.004 |
| 00232 Caffeine metabolism | 1 | BSM | 0.004 |
| 00072 Synthesis and degradation of ketone bodies | 8 | LM | 0.003 |
| 00901 Indole alkaloid biosynthesis | 2 | BSM | 0.003 |
| 00909 Sesquiterpenoid and triterpenoid biosynthesis | 3 | MTP | 0.003 |
| 00591 Linoleic acid metabolism | 6 | LM | 0.003 |
| 00905 Brassinosteroid biosynthesis | 2 | MTP | 0.002 |
| 00196 Photosynthesis - antenna proteins | 3 | EM | 0.001 |
| 00902 Monoterpenoid biosynthesis | 2 | MTP | 0.001 |
| 00965 Betalain biosynthesis | 1 | BSM | 0.000 |
| 00523 Polyketide sugar unit biosynthesis | 1 | MTP | 0.000 |
