# Supplementary material for: Metabolic Pathway Assignment of Plant Genes based on Phylogenetic Profiling–A Feasibility Study
Source: Front Plant Sci. 2017 Oct 27;8:1831. doi: 10.3389/fpls.2017.01831 (PMC5664361; doi:10.3389/fpls.2017.01831)
Supplement: Supplementary file 3 [file Table3.DOCX]

**Supplementary Table 3.** Statistics of metabolism pathway assignments and phylogenetic profile identities of gene families and singletons based on PlantCyc pathway and gene annotation information.

Of all PlantCyc genes, 21,680 PlantCyc genes carrying a pathway annotation could be mapped to Ensembl Plants genes. Mapping was based on protein sequence comparison using BLAST requiring greater than 90% sequence identity, a mismatch count less than 4, and a gap opening count less than 2. This set comprised 17 plant species (species/gene count: ATA/2164, ATH/1666, ATR/1076, BDI/1050, BRP/2630, CRE/351, DOSA/1096, GMX/2450, POP/1549, PPP/1224, SITA/1238, SLY/2048, SMO/1642, SOT/1747, TUR/2386, VVI/1543, ZMA/11 – note that the low number of 11 in ZMA is caused by an ID/sequence database loss of correspondence between our dataset and PlantCyc; see Table 2 for species abbreviation definitions).

For the associated set of 241 PlantCyc metabolism pathways available for analysis, listed are the number of gene families or singleton genes (referred to as gene objects, N_GF/S_) annotated to them, the fraction of all profile-profile comparisons among all N_GF/S_ gene objects yielding identical profiles within a pathway (F_pw_, Eq.1), the fraction of all profile-profile comparisons of N_GF/S_ gene objects yielding identical profiles within and to gene objects outside a pathway (F_all_, Eq.2), the resulting fold enrichment (E= F_pw_/F_all_) of identical phylogenetic profiles within a pathway relative to expectation, and associated Benjamini-Hochberg corrected empirical p-value based on 1,000 random pathway assignments. Results are based on Network30-based gene family assignments (see Methods). Pathways are sorted in ascending order of p-value.

| **PlantCyc pathway name** | **N_GF/S_** | **F_pw_** | **F_all_** | **E = F_pw_/F_all_** | **Adjusted p-value** |
| --- | --- | --- | --- | --- | --- |
| Calvin-Benson-Bassham cycle | 26 | 0.206 | 0.044 | 4.70 | <0.001 |
| brassinosteroids inactivation | 15 | 0.210 | 0.050 | 4.16 | <0.001 |
| oryzalide A biosynthesis | 7 | 0.333 | 0.064 | 5.18 | 0.048 |
| 5-aminoimidazole ribonucleotide biosynthesis II | 11 | 0.182 | 0.043 | 4.26 | 0.048 |
| L-arginine biosynthesis II (acetyl cycle) | 18 | 0.137 | 0.040 | 3.45 | 0.048 |
| L-citrulline biosynthesis | 25 | 0.097 | 0.036 | 2.66 | 0.103 |
| gluconeogenesis I | 51 | 0.064 | 0.029 | 2.16 | 0.103 |
| pyrethrin I biosynthesis | 11 | 0.182 | 0.055 | 3.30 | 0.121 |
| quercetin glycoside biosynthesis (Arabidopsis) | 22 | 0.095 | 0.037 | 2.56 | 0.121 |
| gluconeogenesis III | 56 | 0.064 | 0.032 | 2.00 | 0.121 |
| capsidiol biosynthesis | 6 | 0.267 | 0.067 | 4.00 | 0.132 |
| chorismate biosynthesis from 3-dehydroquinate | 4 | 0.500 | 0.069 | 7.22 | 0.138 |
| malate-oxaloacetate shuttle II | 7 | 0.286 | 0.071 | 4.04 | 0.138 |
| eupatolitin 3-O-glucoside biosynthesis | 19 | 0.099 | 0.040 | 2.51 | 0.138 |
| 2-oxoglutarate decarboxylation to succinyl-CoA | 4 | 0.500 | 0.071 | 7.08 | 0.145 |
| Quercetin glucoside biosynthesis (Allium) | 23 | 0.087 | 0.036 | 2.44 | 0.196 |
| glyoxylate cycle | 15 | 0.105 | 0.041 | 2.57 | 0.227 |
| sinapate ester biosynthesis | 16 | 0.092 | 0.045 | 2.02 | 0.228 |
| indole-3-acetate conjugate biosynthesis II | 2 | 1.000 | 0.050 | 19.92 | 0.241 |
| superpathway of polyamine biosynthesis II | 2 | 1.000 | 0.093 | 10.79 | 0.241 |
| 4-aminobutanoate degradation IV | 6 | 0.200 | 0.060 | 3.33 | 0.241 |
| L-arginine biosynthesis I (via L-ornithine) | 9 | 0.167 | 0.044 | 3.82 | 0.241 |
| superpathway of anthocyanin biosynthesis | 11 | 0.127 | 0.040 | 3.17 | 0.241 |
| L-lysine biosynthesis VI | 15 | 0.105 | 0.044 | 2.36 | 0.241 |
| lysine degradation II | 18 | 0.085 | 0.047 | 1.79 | 0.241 |
| Phenylmethanethial S-oxide biosynthesis | 2 | 1.000 | 0.050 | 19.92 | 0.269 |
| pyrimidine deoxyribonucleotides dephosphorylation | 11 | 0.109 | 0.042 | 2.59 | 0.295 |
| indole-3-acetate degradation II | 2 | 1.000 | 0.050 | 19.92 | 0.310 |
| UMP biosynthesis I | 6 | 0.200 | 0.049 | 4.05 | 0.324 |
| glycolysis I (from glucose 6-phosphate) | 54 | 0.050 | 0.028 | 1.76 | 0.337 |
| xyloglucan biosynthesis | 7 | 0.143 | 0.037 | 3.86 | 0.351 |
| pinobanksin biosynthesis | 10 | 0.089 | 0.030 | 3.00 | 0.351 |
| L-aspartate degradation I | 6 | 0.133 | 0.051 | 2.60 | 0.351 |
| L-aspartate biosynthesis | 6 | 0.133 | 0.051 | 2.60 | 0.351 |
| diterpene phytoalexins precursors biosynthesis | 6 | 0.133 | 0.052 | 2.55 | 0.351 |
| scopoletin biosynthesis | 7 | 0.143 | 0.030 | 4.75 | 0.362 |
| 4-aminobutanoate degradation I | 7 | 0.143 | 0.052 | 2.77 | 0.393 |
| linalool biosynthesis I | 7 | 0.143 | 0.052 | 2.73 | 0.393 |
| vicianin bioactivation | 3 | 0.333 | 0.042 | 7.95 | 0.408 |
| levopimaric acid biosynthesis | 3 | 0.333 | 0.035 | 9.61 | 0.413 |
| isopimaric acid biosynthesis | 3 | 0.333 | 0.035 | 9.61 | 0.413 |
| amygdalin and prunasin degradation | 3 | 0.333 | 0.042 | 7.95 | 0.413 |
| L-alanine biosynthesis II | 3 | 0.333 | 0.050 | 6.70 | 0.413 |
| &beta;-caryophyllene biosynthesis | 3 | 0.333 | 0.054 | 6.14 | 0.413 |
| cyanidin dimalonylglucoside biosynthesis | 3 | 0.333 | 0.076 | 4.40 | 0.413 |
| superpathway of pyrimidine ribonucleosides salvage | 3 | 0.333 | 0.079 | 4.23 | 0.413 |
| artemisinin biosynthesis | 3 | 0.333 | 0.079 | 4.20 | 0.413 |
| pyrimidine nucleobases salvage I | 3 | 0.333 | 0.079 | 4.23 | 0.413 |
| abietic acid biosynthesis | 8 | 0.107 | 0.042 | 2.56 | 0.413 |
| methylerythritol phosphate pathway II | 13 | 0.077 | 0.031 | 2.49 | 0.413 |
| volatile benzenoid biosynthesis I (ester formation) | 12 | 0.076 | 0.036 | 2.10 | 0.413 |
| quercetin gentiotetraside biosynthesis | 18 | 0.059 | 0.031 | 1.90 | 0.413 |
| flavonol biosynthesis | 8 | 0.107 | 0.029 | 3.68 | 0.432 |
| L-homocysteine and L-cysteine interconversion | 3 | 0.333 | 0.078 | 4.27 | 0.433 |
| glucosinolate biosynthesis from dihomomethionine | 11 | 0.073 | 0.031 | 2.34 | 0.447 |
| flavonol glucosylation I | 18 | 0.059 | 0.031 | 1.90 | 0.504 |
| all-trans-farnesol biosynthesis | 9 | 0.083 | 0.042 | 2.00 | 0.536 |
| octanoyl-[acyl-carrier protein] biosynthesis | 16 | 0.058 | 0.033 | 1.77 | 0.536 |
| geranyl diphosphate biosynthesis | 4 | 0.167 | 0.048 | 3.46 | 0.564 |
| UDP-sugars interconversion | 4 | 0.167 | 0.048 | 3.51 | 0.574 |
| kaempferol gentiobioside biosynthesis | 12 | 0.061 | 0.034 | 1.80 | 0.597 |
| polyisoprenoid biosynthesis | 4 | 0.167 | 0.048 | 3.46 | 0.610 |
| 1,3,5-trimethoxybenzene biosynthesis | 8 | 0.071 | 0.041 | 1.76 | 0.620 |
| aerobic respiration I (cytochrome c) | 103 | 0.035 | 0.022 | 1.59 | 0.674 |
| ornithine-citrulline shuttle | 10 | 0.067 | 0.034 | 1.97 | 0.682 |
| 4-aminobenzoate biosynthesis | 5 | 0.100 | 0.037 | 2.69 | 0.778 |
| gentiodelphin biosynthesis | 5 | 0.100 | 0.039 | 2.59 | 0.778 |
| genistein conjugates interconversion | 9 | 0.056 | 0.037 | 1.52 | 0.778 |
| chlorogenic acid biosynthesis I | 13 | 0.051 | 0.033 | 1.56 | 0.778 |
| pyrimidine deoxyribonucleotides de novo biosynthesis I | 20 | 0.042 | 0.036 | 1.16 | 0.778 |
| luteolinidin 5-O-glucoside biosynthesis | 5 | 0.100 | 0.032 | 3.14 | 0.799 |
| L-cysteine biosynthesis I | 5 | 0.100 | 0.047 | 2.13 | 0.799 |
| homogalacturonan degradation | 26 | 0.040 | 0.019 | 2.09 | 0.799 |
| violdelphin biosynthesis | 5 | 0.100 | 0.048 | 2.09 | 0.801 |
| anthocyanidin modification (Arabidopsis) | 9 | 0.056 | 0.031 | 1.81 | 0.813 |
| flavonoid biosynthesis (in equisetum) | 14 | 0.044 | 0.021 | 2.08 | 0.831 |
| salicortin biosynthesis | 12 | 0.045 | 0.046 | 0.98 | 0.880 |
| syringetin biosynthesis | 6 | 0.067 | 0.032 | 2.10 | 0.916 |
| 6-hydroxymethyl-dihydropterin diphosphate biosynthesis I | 6 | 0.067 | 0.033 | 2.03 | 0.916 |
| lysine degradation III | 6 | 0.067 | 0.049 | 1.36 | 0.916 |
| cyanide detoxification I | 6 | 0.067 | 0.053 | 1.25 | 0.916 |
| superpathway of plastoquinol biosynthesis | 6 | 0.067 | 0.052 | 1.28 | 0.916 |
| 4-hydroxybenzoate biosynthesis I (eukaryotes) | 10 | 0.044 | 0.036 | 1.25 | 0.916 |
| L-tryptophan biosynthesis | 18 | 0.039 | 0.034 | 1.17 | 0.916 |
| ethanol degradation IV | 6 | 0.067 | 0.063 | 1.05 | 0.916 |
| very long chain fatty acid biosynthesis II | 28 | 0.034 | 0.034 | 1.02 | 0.916 |
| pyruvate fermentation to ethanol I | 6 | 0.067 | 0.033 | 2.01 | 0.956 |
| pyridine nucleotide cycling (plants) | 15 | 0.038 | 0.022 | 1.77 | 0.980 |
| lotaustralin biosynthesis | 2 | 0.000 | 0.000 |  | 1.000 |
| luteolin glycosides biosynthesis | 2 | 0.000 | 0.000 |  | 1.000 |
| diphthamide biosynthesis (archaea) | 2 | 0.000 | 0.000 |  | 1.000 |
| NADH repair | 2 | 0.000 | 0.000 |  | 1.000 |
| phosphatidylglycerol biosynthesis II (non-plastidic) | 7 | 0.000 | 0.000 |  | 1.000 |
| rose anthocyanin biosynthesis II (via cyanidin 3-O-&beta;-D-glucoside) | 7 | 0.048 | 0.018 | 2.70 | 1.000 |
| sucrose degradation III (sucrose invertase) | 16 | 0.033 | 0.012 | 2.72 | 1.000 |
| vitamin E biosynthesis (tocopherols) | 7 | 0.048 | 0.022 | 2.18 | 1.000 |
| betacyanin biosynthesis | 7 | 0.048 | 0.035 | 1.35 | 1.000 |
| fatty acid beta-oxidation V | 7 | 0.048 | 0.040 | 1.20 | 1.000 |
| caffeoylglucarate biosynthesis | 8 | 0.036 | 0.028 | 1.29 | 1.000 |
| methylglyoxal degradation I | 8 | 0.036 | 0.030 | 1.18 | 1.000 |
| flavonoid biosynthesis | 19 | 0.023 | 0.019 | 1.20 | 1.000 |
| urate biosynthesis/inosine 5-phosphate degradation | 8 | 0.036 | 0.033 | 1.07 | 1.000 |
| phosphatidate metabolism, as a signaling molecule | 14 | 0.022 | 0.020 | 1.12 | 1.000 |
| thioredoxin pathway | 7 | 0.048 | 0.044 | 1.08 | 1.000 |
| trans-lycopene biosynthesis II (plants) | 13 | 0.026 | 0.024 | 1.08 | 1.000 |
| Organic Nitrogen Assimilation | 22 | 0.017 | 0.018 | 0.99 | 1.000 |
| fatty acid elongation -- saturated | 9 | 0.028 | 0.027 | 1.02 | 1.000 |
| coumarins biosynthesis (engineered) | 13 | 0.013 | 0.013 | 0.97 | 1.000 |
| 3,8-divinyl-chlorophyllide a biosynthesis I (aerobic, light-dependent) | 15 | 0.029 | 0.029 | 0.98 | 1.000 |
| superpathway of fermentation (Chlamydomonas reinhardtii) | 11 | 0.018 | 0.019 | 0.97 | 1.000 |
| secologanin and strictosidine biosynthesis | 20 | 0.026 | 0.029 | 0.90 | 1.000 |
| DIBOA-glucoside biosynthesis | 11 | 0.036 | 0.042 | 0.86 | 1.000 |
| fatty acid biosynthesis initiation I | 10 | 0.022 | 0.029 | 0.77 | 1.000 |
| phosphatidate biosynthesis (yeast) | 16 | 0.017 | 0.020 | 0.83 | 1.000 |
| palmitate biosynthesis II (bacteria and plants) | 21 | 0.024 | 0.032 | 0.75 | 1.000 |
| jasmonic acid biosynthesis | 13 | 0.026 | 0.036 | 0.71 | 1.000 |
| (S)-reticuline biosynthesis I | 8 | 0.036 | 0.050 | 0.72 | 1.000 |
| CDP-diacylglycerol biosynthesis I | 17 | 0.015 | 0.022 | 0.66 | 1.000 |
| D-galactose degradation I (Leloir pathway) | 11 | 0.018 | 0.028 | 0.66 | 1.000 |
| superpathway of aspartate and asparagine biosynthesis | 14 | 0.022 | 0.035 | 0.63 | 1.000 |
| methyl ketone biosynthesis (engineered) | 17 | 0.022 | 0.037 | 0.60 | 1.000 |
| very long chain fatty acid biosynthesis I | 28 | 0.013 | 0.022 | 0.59 | 1.000 |
| phosphatidylcholine acyl editing | 37 | 0.008 | 0.017 | 0.43 | 1.000 |
| L-asparagine degradation I | 5 | 0.000 | 0.032 | 0.00 | 1.000 |
| choline degradation I | 2 | 0.000 | 0.072 | 0.00 | 1.000 |
| coenzyme A biosynthesis I | 7 | 0.000 | 0.016 | 0.00 | 1.000 |
| glutathione-glutaredoxin redox reactions | 3 | 0.000 | 0.064 | 0.00 | 1.000 |
| xylitol degradation | 4 | 0.000 | 0.000 | 0.00 | 1.000 |
| protein N-glycosylation (eukaryotic, high mannose) | 21 | 0.000 | 0.014 | 0.00 | 1.000 |
| superpathway of phospholipid biosynthesis II (plants) | 7 | 0.000 | 0.021 | 0.00 | 1.000 |
| pyridoxal 5-phosphate salvage I PNPOXI-RXN EC-1.4.3.5 G2Z-12331-MONOMER pyridoxine 5-phosphate oxidase | 4 | 0.000 | 0.006 | 0.00 | 1.000 |
| ppGpp biosynthesis | 4 | 0.000 | 0.040 | 0.00 | 1.000 |
| L-proline biosynthesis I | 2 | 0.000 | 0.047 | 0.00 | 1.000 |
| plastoquinol-9 biosynthesis I | 5 | 0.000 | 0.011 | 0.00 | 1.000 |
| isoflavonoid biosynthesis II | 5 | 0.000 | 0.045 | 0.00 | 1.000 |
| free phenylpropanoid acid biosynthesis | 4 | 0.000 | 0.017 | 0.00 | 1.000 |
| formononetin biosynthesis | 3 | 0.000 | 0.052 | 0.00 | 1.000 |
| brassinosteroid biosynthesis II | 2 | 0.000 | 0.072 | 0.00 | 1.000 |
| trans-zeatin biosynthesis | 8 | 0.000 | 0.035 | 0.00 | 1.000 |
| alkane oxidation | 4 | 0.000 | 0.075 | 0.00 | 1.000 |
| choline biosynthesis III | 12 | 0.000 | 0.019 | 0.00 | 1.000 |
| glycerol degradation I | 4 | 0.000 | 0.024 | 0.00 | 1.000 |
| lipid-dependent phytate biosynthesis I (via Ins(1,4,5)P<sub>3</sub>) | 3 | 0.000 | 0.005 | 0.00 | 1.000 |
| arsenate detoxification II (glutaredoxin) | 5 | 0.000 | 0.012 | 0.00 | 1.000 |
| ent-kaurene biosynthesis I | 5 | 0.000 | 0.042 | 0.00 | 1.000 |
| S-adenosyl-L-methionine cycle II | 9 | 0.000 | 0.021 | 0.00 | 1.000 |
| rosmarinic acid biosynthesis II | 6 | 0.000 | 0.039 | 0.00 | 1.000 |
| luteolin biosynthesis | 3 | 0.000 | 0.021 | 0.00 | 1.000 |
| chlorophyll cycle | 5 | 0.000 | 0.034 | 0.00 | 1.000 |
| superpathway of geranylgeranyl diphosphate biosynthesis II (via MEP) | 5 | 0.000 | 0.034 | 0.00 | 1.000 |
| pelargonidin conjugates biosynthesis | 2 | 0.000 | 0.078 | 0.00 | 1.000 |
| cannabinoid biosynthesis | 10 | 0.000 | 0.026 | 0.00 | 1.000 |
| acyl-CoA hydrolysis | 2 | 0.000 | 0.072 | 0.00 | 1.000 |
| soybean saponin I biosynthesis | 6 | 0.000 | 0.031 | 0.00 | 1.000 |
| phaseic acid biosynthesis | 2 | 0.000 | 0.025 | 0.00 | 1.000 |
| shisonin biosynthesis | 3 | 0.000 | 0.058 | 0.00 | 1.000 |
| sanguinarine and macarpine biosynthesis | 6 | 0.000 | 0.023 | 0.00 | 1.000 |
| astaxanthin biosynthesis (bacteria, fungi, algae) | 3 | 0.000 | 0.052 | 0.00 | 1.000 |
| arachidonate biosynthesis I (6-desaturase, lower eukaryotes) | 2 | 0.000 | 0.004 | 0.00 | 1.000 |
| superpathway of betalain biosynthesis | 5 | 0.000 | 0.031 | 0.00 | 1.000 |
| divinyl ether biosynthesis I | 2 | 0.000 | 0.007 | 0.00 | 1.000 |
| 9-lipoxygenase and 9-allene oxide synthase pathway | 2 | 0.000 | 0.007 | 0.00 | 1.000 |
| (3E)-4,8-dimethylnona-1,3,7-triene biosynthesis | 4 | 0.000 | 0.044 | 0.00 | 1.000 |
| S-methyl-L-methionine cycle | 3 | 0.000 | 0.015 | 0.00 | 1.000 |
| matairesinol biosynthesis | 7 | 0.000 | 0.010 | 0.00 | 1.000 |
| lupanine biosynthesis | 3 | 0.000 | 0.030 | 0.00 | 1.000 |
| pyruvate fermentation to ethanol II | 6 | 0.000 | 0.043 | 0.00 | 1.000 |
| sorbitol biosynthesis II | 3 | 0.000 | 0.001 | 0.00 | 1.000 |
| phosphatidylethanolamine biosynthesis I | 6 | 0.000 | 0.024 | 0.00 | 1.000 |
| urate degradation to allantoin I | 5 | 0.000 | 0.008 | 0.00 | 1.000 |
| gossypol biosynthesis | 2 | 0.000 | 0.009 | 0.00 | 1.000 |
| xylan biosynthesis | 3 | 0.000 | 0.017 | 0.00 | 1.000 |
| usnate biosynthesis | 2 | 0.000 | 0.007 | 0.00 | 1.000 |
| geraniol and geranial biosynthesis | 6 | 0.000 | 0.051 | 0.00 | 1.000 |
| wax esters biosynthesis I | 9 | 0.000 | 0.005 | 0.00 | 1.000 |
| glutaminyl-tRNA<sup>gln</sup> biosynthesis via transamidation | 6 | 0.000 | 0.020 | 0.00 | 1.000 |
| (4R)-carvone biosynthesis | 3 | 0.000 | 0.058 | 0.00 | 1.000 |
| zeaxanthin, antheraxanthin and violaxanthin interconversion | 5 | 0.000 | 0.000 | 0.00 | 1.000 |
| &delta;-carotene biosynthesis | 2 | 0.000 | 0.001 | 0.00 | 1.000 |
| Fe(II)-nicotianamine transport in phloem | 2 | 0.000 | 0.004 | 0.00 | 1.000 |
| pseudouridine degradation | 4 | 0.000 | 0.017 | 0.00 | 1.000 |
| chlorogenic acid biosynthesis II | 2 | 0.000 | 0.025 | 0.00 | 1.000 |
| dimethylsulfoniopropanoate biosynthesis II (Spartina) | 2 | 0.000 | 0.021 | 0.00 | 1.000 |
| methylquercetin biosynthesis | 2 | 0.000 | 0.009 | 0.00 | 1.000 |
| botryococcenes and methylated squalene biosynthesis | 2 | 0.000 | 0.047 | 0.00 | 1.000 |
| mangrove triterpenoid biosynthesis | 2 | 0.000 | 0.001 | 0.00 | 1.000 |
| inosine-5-phosphate biosynthesis IIAIRCARBOXY-RXNEC-4.1.1.21G30-8981-MONOMERphosphoribosylaminoimidazole carboxylaseG30-8981Bra005171.1-P PWY-6124inosine-5-phosphate biosynthesis II | 5 | 0.000 | 0.020 | 0.00 | 1.000 |
| jasmonoyl-amino acid conjugates biosynthesis I | 2 | 0.000 | 0.025 | 0.00 | 1.000 |
| tuberonate glucoside biosynthesis | 2 | 0.000 | 0.025 | 0.00 | 1.000 |
| D-myo-inositol (1,4,5)-trisphosphate biosynthesis | 25 | 0.000 | 0.016 | 0.00 | 1.000 |
| D-myo-inositol (1,3,4)-trisphosphate biosynthesis | 4 | 0.000 | 0.043 | 0.00 | 1.000 |
| hispidol and hispidol 4-O-&beta;-D-glucoside biosynthesisRXN-11084EC-1.11.2GN7V-55220-MONOMERGlyma.15G129200.1.pGN7V-55220Glyma.15G129200.1.p PWY-6401hispidol and hispidol 4-O-&beta;-D-glucoside biosynthesis | 3 | 0.000 | 0.010 | 0.00 | 1.000 |
| spermine and spermidine degradation III | 3 | 0.000 | 0.017 | 0.00 | 1.000 |
| trans-cinnamoyl-CoA biosynthesis | 6 | 0.000 | 0.007 | 0.00 | 1.000 |
| pyridoxal 5-phosphate biosynthesis II RXN-11322 EC-4.3.3.6 G5X-5045-MONOMER pyridoxal 5-phosphate synthase | 2 | 0.000 | 0.002 | 0.00 | 1.000 |
| ceramide degradation | 4 | 0.000 | 0.047 | 0.00 | 1.000 |
| C-glycosylflavone biosynthesis I | 2 | 0.000 | 0.010 | 0.00 | 1.000 |
| O -methylation of tricetin | 4 | 0.000 | 0.043 | 0.00 | 1.000 |
| plant sterol biosynthesis II | 3 | 0.000 | 0.017 | 0.00 | 1.000 |
| (E,E)-4,8,12-trimethyltrideca-1,3,7,11-tetraene biosynthesis | 2 | 0.000 | 0.002 | 0.00 | 1.000 |
| plaunotol biosynthesis | 5 | 0.000 | 0.034 | 0.00 | 1.000 |
| S-methyl-5-thioadenosine degradation I5-METHYLTHIORIBOSE-KINASE-RXNEC-2.7.1.100G3F-8565-MONOMERSolyc01g107550.3.1G3F-8565Solyc01g107550.3.1 PWY-6754S-methyl-5-thioadenosine degradation I | 3 | 0.000 | 0.001 | 0.00 | 1.000 |
| salicylate glucosides biosynthesis IV | 5 | 0.000 | 0.035 | 0.00 | 1.000 |
| thiamine salvage III | 5 | 0.000 | 0.012 | 0.00 | 1.000 |
| chlorophyll a degradation II | 6 | 0.000 | 0.041 | 0.00 | 1.000 |
| phenolic malonylglucosides biosynthesis | 3 | 0.000 | 0.052 | 0.00 | 1.000 |
| brassinosteroid biosynthesis I | 2 | 0.000 | 0.072 | 0.00 | 1.000 |
| steviol glucoside biosynthesis (rebaudioside A biosynthesis) | 3 | 0.000 | 0.007 | 0.00 | 1.000 |
| esterified suberin biosynthesis | 4 | 0.000 | 0.018 | 0.00 | 1.000 |
| noscapine biosynthesis | 4 | 0.000 | 0.014 | 0.00 | 1.000 |
| sesaminol glucoside biosynthesis | 3 | 0.000 | 0.006 | 0.00 | 1.000 |
| 7-dehydroporiferasterol biosynthesis | 4 | 0.000 | 0.015 | 0.00 | 1.000 |
| polymethylated myricetin biosynthesis (tomato) | 2 | 0.000 | 0.009 | 0.00 | 1.000 |
| pyrimidine deoxyribonucleosides salvage | 5 | 0.000 | 0.005 | 0.00 | 1.000 |
| pyridoxal 5-phosphate salvage II (plants)3.1.3.74-RXNEC-3.1.3.74GN7V-65716-MONOMERpyridoxal phosphataseGN7V-65716Glyma.07G206300.1.p PWY-7204pyridoxal 5-phosphate salvage II (plants) | 6 | 0.000 | 0.002 | 0.00 | 1.000 |
| purine deoxyribonucleosides salvage | 6 | 0.000 | 0.023 | 0.00 | 1.000 |
| guanosine deoxyribonucleotides de novo biosynthesis I | 2 | 0.000 | 0.059 | 0.00 | 1.000 |
| pelargonidin diglucoside biosynthesis (acyl-glucose dependent) | 3 | 0.000 | 0.052 | 0.00 | 1.000 |
| ternatin C3 biosynthesis | 2 | 0.000 | 0.078 | 0.00 | 1.000 |
| salvigenin biosynthesis | 4 | 0.000 | 0.016 | 0.00 | 1.000 |
| papaverine biosynthesis | 3 | 0.000 | 0.003 | 0.00 | 1.000 |
| urate degradation to allantoin II | 4 | 0.000 | 0.002 | 0.00 | 1.000 |
| phenylpropanoids methylation (ice plant) | 2 | 0.000 | 0.009 | 0.00 | 1.000 |
| phosphatidylinositol biosynthesis II (eukaryotes) | 4 | 0.000 | 0.024 | 0.00 | 1.000 |
| anthocyanidin acylglucoside and acylsambubioside biosynthesis | 3 | 0.000 | 0.052 | 0.00 | 1.000 |
| carnosate bioynthesis | 3 | 0.000 | 0.001 | 0.00 | 1.000 |
| glycolipid desaturation | 5 | 0.000 | 0.032 | 0.00 | 1.000 |
| Amaryllidacea alkaloids biosynthesis | 3 | 0.000 | 0.004 | 0.00 | 1.000 |
| resveratrol biosynthesis | 2 | 0.000 | 0.013 | 0.00 | 1.000 |
| acetate formation from acetyl-CoA I | 2 | 0.000 | 0.001 | 0.00 | 1.000 |
| L-lysine degradation I | 2 | 0.000 | 0.003 | 0.00 | 1.000 |
| PRPP biosynthesis I | 2 | 0.000 | 0.047 | 0.00 | 1.000 |
| L-ascorbate biosynthesis IV | 3 | 0.000 | 0.026 | 0.00 | 1.000 |
| phospholipases (Chlamydomonas) | 6 | 0.000 | 0.032 | 0.00 | 1.000 |
| sulfoquinovosyl diacylglycerol biosynthesis | 4 | 0.000 | 0.013 | 0.00 | 1.000 |
| glutathione degradation | 4 | 0.000 | 0.004 | 0.00 | 1.000 |
| pyrimidine salvage pathway | 2 | 0.000 | 0.047 | 0.00 | 1.000 |
| indole glucosinolate activation (herbivore attack) | 4 | 0.000 | 0.052 | 0.00 | 1.000 |
| indole glucosinolate activation (intact plant cell) | 4 | 0.000 | 0.034 | 0.00 | 1.000 |
| CDP-diacylglycerol biosynthesis IV | 10 | 0.000 | 0.014 | 0.00 | 1.000 |
| trehalose biosynthesis I | 5 | 0.000 | 0.039 | 0.00 | 1.000 |
